# Synthesis and Characterization of Ligand-Stabilized Silver Nanoparticles and Comparative Antibacterial Activity against *E. coli*

**DOI:** 10.3390/ijms232315251

**Published:** 2022-12-03

**Authors:** Nishal M. Egodawaththa, Amy L. Knight, Jingxuan Ma, D. Andrew Knight, Eric Guisbert, Nasri Nesnas

**Affiliations:** Department of Biomedical and Chemical Engineering and Sciences, Florida Institute of Technology, Melbourne, FL 32901, USA

**Keywords:** silver, *Escherichia coli*, antibacterial activity, nanoparticle

## Abstract

Silver is a well-established antimicrobial agent. Conjugation of organic ligands with silver nanoparticles has been shown to create antimicrobial nanoparticles with improved pharmacodynamic properties and reduced toxicity. Twelve novel organic ligand functionalized silver nanoparticles (AgNPs) were prepared via a light-controlled reaction with derivatives of benzothiazole, benzoxazine, quinazolinone, 2-butyne-1,4-diol, 3-butyne-1-ol, and heptane-1,7-dioic. UV-vis, Fourier-transform infrared (FTIR) spectroscopy, and energy-dispersive X-ray (EDAX) analysis were used to confirm the successful formation of ligand-functionalized nanoparticles. Dynamic light scattering (DLS) revealed mean nanoparticle diameters between 25 and 278 nm. Spherical and nanotube-like morphologies were observed using transmission electron microscopy (TEM) and scanning electron microscopy (SEM). Seven of the twelve nanoparticles exhibited strong antimicrobial activity and five of the twelve demonstrated significant antibacterial capabilities against *E. coli* in a zone-of-inhibition assay. The synthesis of functionalized silver nanoparticles such as the twelve presented is critical for the further development of silver-nanoconjugated antibacterial agents.

## 1. Introduction

Silver is a toxic transition metal and a known antibacterial agent against both aerobic and anaerobic bacteria [1,2]. It has been incorporated into wound dressings and medical treatments for centuries. More recently, nanotechnology has amplified the efficiency of silver in medicine [3,4]. Silver nanoparticles have a high surface area-to-volume ratio and unique chemical and physical properties, making them ideal for antibacterial use [5]. Specifically, they have been shown to inhibit bacterial growth through mechanisms that include the precipitation of cellular proteins, interference with DNA function, and inhibition of the electron transport chain [6]. Silver nanoparticles also demonstrate antibacterial effects in both Gram-positive and Gram-negative bacteria [7].

Several distinct silver nanoparticles have been synthesized for medicinal use in the last decade. However, many of the syntheses are limited by low yields and therefore rendered less useful while the need for new antibacterial nanoparticles remains. Recently, AgNPs synthesized with quinazolin-4(3*H*)-one derivatives were developed by Abdulkader Masri’s group [8]. This work has provided a foundation for new organic ligand-functionalized AgNPs to be further developed.

One promising route being studied currently for improving the synthesis and efficacy of silver nanoparticles is the incorporation of organic compounds. For example, quinazolinone is a highly stable nitrogen-containing heterocyclic scaffold used to generate antibacterial drugs [8,9]. Benzoxazine is a bicyclic compound containing an oxazine ring attached to a benzene ring that acts as a basis for the synthesis of other organic ligands such as quinazolinone [10,11]. Both benzothiazole, a benzene ring fused with a thiazole ring, and heptane-1,7-dioic, commonly referred to as pimelic acid, are also used as starting materials for antibacterial compounds [12,13]. These organic ligands can be coated with silver, enabling their conjugation with nanoparticles.

**Figure 1:**
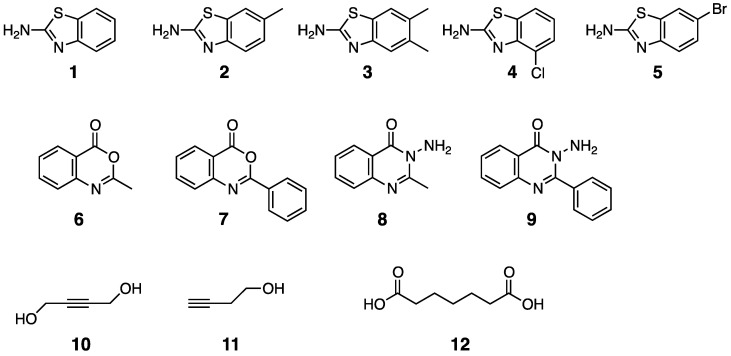
Molecular structures of the organic ligands used (**1**: 2-aminobenzothiazole, **2**: 5-methyl-aminobenzothiazole, **3**: 5,6-dimethyl-2-aminobenzothiazole, **4**: 2-amino-4-chlorobenzothiazole, **5**: 2-amino-6-bromobenzothiazole, **6**: 2-methyl-4*H*-3,1-benzoxazin-4-one, **7**: 2-phenyl-4*H*-3,1-benzoxazin-4-one, **8**: 3-amino-2-methyl-4(3*H*)-quinazolinone, **9**: 3-amino-2-phenyl-4(3*H*)-quinazolinone, **10**: 2-butyne-1,4-diol, **11**: 3-butyn-1-ol, **12**: heptane-1,7-dioic).

Herein, we present the syntheses and characterizations of 12 conjugated silver nanoparticles using red light (620 nm) irradiation with a variety of organic ligands. These nanoparticles were prepared using derivatives of benzothiazole (**1**–**5**), benzoxazine (**6** and **7**), quinazolinone (**8** and **9**), 2-butyne-1,4-diol (**10**), 3-butyn-1-ol (**11**), and heptane-1,7-dioic (**12**), shown in Figure 1. The 12 organic ligand-conjugated AgNPs were developed with the hypothesis that different properties of the organic ligands used would influence antimicrobial activity, leading to a range of AgNPs with various antibacterial capabilities. The resulting AgNPs were characterized by several methods to confirm their formation and determine their properties. Their antimicrobial properties were then tested against *E. coli* to evaluate the validity of the hypothesis and determine the potential of the 12 AgNPs for use as antimicrobial agents.

## 2. Results

Twelve organic ligand functionalized silver nanoparticles (AgNPs) were synthesized and characterized with UV-vis spectroscopy, FTIR, EDAX, zeta potential analysis, TEM, SEM, and DLS analysis to confirm the formation, size, and shape of the nanoparticles.

### 2.1. UV-Vis and FTIR Spectroscopy

**Figure 2:**
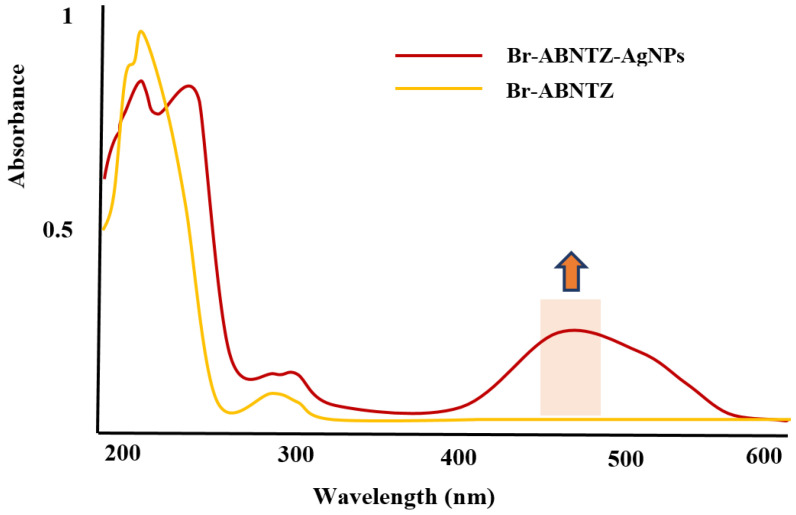
Representative UV-vis spectra of functionalized 2-amino-6-bromobenzothiazole–AgNPs (**5-np**) (shown in red) with the corresponding organic ligand **5** (shown in yellow). The nanoconjugate displayed a characteristic SPR band maximum within the typical range of 380–480 nm.

The UV-vis spectra of the organic ligands (**1**–**12**) were measured both independently and again following conjugation with AgNPs. The presence of SPR maxima within the typical range of 380–480 nm confirmed successful nanoparticle formation [14]. In Figure 2, the comparative UV-vis spectra display an SPR maximum of around 460 nm for 2-amino-6-bromobenzothiazole-AgNPs (**5-np**) and no peak at all for its independent organic ligand counterpart. Since the peak present in the AgNPs falls within the characteristic 380–480 nm range and there is no similar peak in the UV-vis spectrum of the isolated organic ligand, successful conjugation of the nanoparticle is apparent. Analyses of the UV-vis spectra for all conjugated silver nanoparticles with their organic ligands can be found in the Supporting Information (Figure S1). Peaks within the characteristic 380–480 nm range were visible for all 12 molecules with similarly missing peaks within that range for the independent organic ligands. This signifies the successful formation of all 12 organic ligand-functionalized AgNPs. Both UV band broadening and red shifting were also observed and can be attributed to some aggregation and size confinement with the size increase of the AgNPs. This may be the result of less confined charge carrier wave functions [14]. Initially, some peaks also showed aggregation due to size variation and difficulty controlling the combination of nanoparticles; however, we were able to overcome the agglomeration by adjusting the dilution parameters of the conjugated silver nanoparticles.

**Figure 3:**
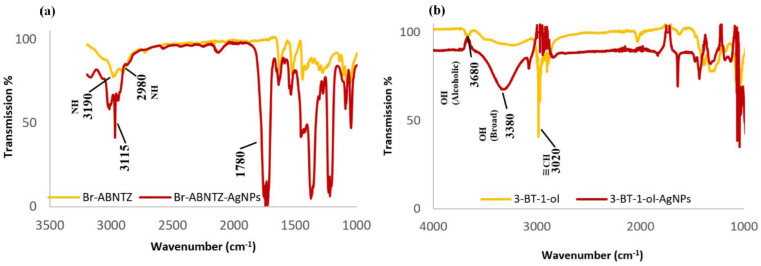
Representative FTIR (Thermo Fisher Scientific MicromATR Vision^TM^ Nicolet IS-5) spectra of (**a**) (**5-np**) (shown in red) and corresponding organic ligand **5** (shown in yellow) along with (**b**) 3-butyn-1-ol-AgNPs (**11-np**) (shown in red) and corresponding organic ligand **11** (shown in yellow).

Additionally, FTIR analyses of the 12 conjugated nanoparticles with their corresponding organic ligands were carried out. The complete set of results can be found in the Supporting Information (Figure S2). The differences between the organic ligands as independent compounds and as nanoparticle conjugates were highly apparent in the FTIR spectra. This can especially be seen in the comparative FTIR analyses of **5-np** and organic ligand **5** along with **11-np** and organic ligand **11**, as shown in Figure 3. All observed bands with slightly varied shifts can be attributed to the distinct vibrational stretching of newly formed functional groups, such as N-H, C=O, C-N, and C=N. This clear functional group transformation was present in the comparative FTIR spectra for all 12 AgNPs and their corresponding organic ligands, further confirming the successful formation of the organic ligand functionalized AgNPs. These results signify that NaBH_4_, the reducing agent used in the synthesis of the AgNPs, most likely led to a functional group transformation that promoted silver coordination with the organic ligands. The organic ligands could have also acted as capping or stabilizing agents for the AgNPs.

Specifically, numerous deviations between **5-np** and organic ligand **5** (Figure 3) were observed. As an example, organic ligand **5** showed two bands at 3190 and 2980 cm^−1^. These data are characteristic of a primary amine (NH_2_) group stretching. Organic ligand **5** possesses an amino-benzothiazole scaffold, but when the organic ligand is conjugated with silver, the band indicating NH_2_ disappears, and one strong band appears at 3115 cm^−1^, as shown by the FTIR spectrum of **5-np**. Also, in the **11-np** to organic ligand **11** FTIR comparison, a minor alcohol peak is apparent at 3680 cm^−1^ for the ligand, but a strong broadband can be observed at 3380 cm^−1^ for the conjugated nanoparticle. This shift could be due to the remote effects of the typical interaction between silver and the terminal alkyne of the organic ligand in the AgNPs. These structural changes indicate the successful formation of the conjugated AgNPs.

### 2.2. EDAX Analysis

**Figure 4:**
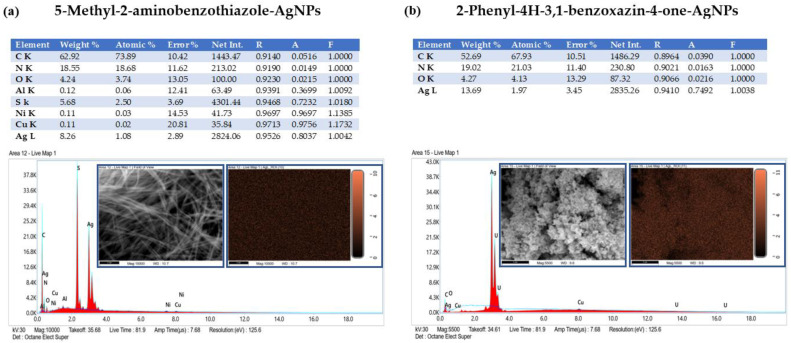
Representative EDAX analyses of (**a**) 5-methyl-2-aminobenzothiazole-AgNPs (**2-np**) and (**b**) 2-phenyl-4H-3,1-benzoxazin-4-one-AgNPs (**7-np**).

Scanning electron microscopy (SEM) with energy-dispersive X-ray analysis (EDAX) was used to further confirm the formations of the conjugated AgNPs. The data generated by EDAX analysis yielded spectra showing peaks corresponding to the elements composing the conjugated AgNPs. This elemental analysis further confirmed the binding of the nanoparticles to the organic ligands. Representative EDAX analyses are shown in Figure 4 and data for all 12 AgNPs can be found in the Supporting Information (Figures S3–S6).

### 2.3. Zeta Potential and Size Measurement

**Figure 5:**
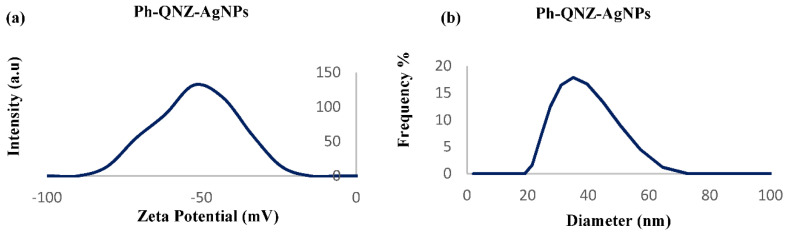
Representative (**a**) zeta potential plot and (**b**) size distribution histogram of 3-amino-2-phenyl-4(3*H*)-quinazolinone-AgNPs (**9-np**) obtained using a Horiba Scientific Nanoparticle Analyzer SZ-100 (all nanoparticles had mean diameters between 25 and 278 nm and a negative surface charge).

Zeta potential values indicate nanoparticle stability, and those of higher magnitude are representative of higher particle stability. Based on the zeta potentials obtained, nine of the twelve AgNPs were considered to have good stability, with zeta potentials around −50 mV. This reflects high electrostatic repulsions between adjacent particles. The 2-butyne-1,4-diol-AgNPs (**10-np**), 3-butyn-1-ol-AgNPs (**11-np**), and heptane-1,7-dioic-AgNPs (**12-np**) showed zeta potentials of −21.4, −19.4, and −16.3 mV, respectively, suggesting poor comparative stability to those of the other AgNPs mentioned in Table 1. These results are displayed by the representative zeta potential graphs in Figure 5 and in full by the values in Table 1. The average sizes of the functionalized nanoparticles were also measured and ranged from 25 to 278 nm, confirming size within the nano range. Size information for the AgNPs can also be found in Table 1.

### 2.4. TEM and SEM Analyses

**Figure 6:**
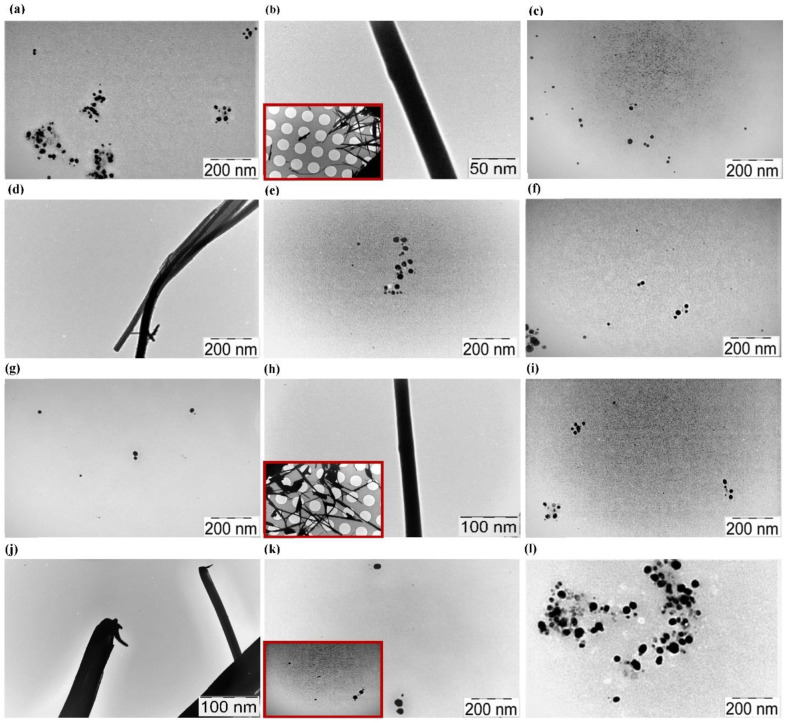
TEM (Zeiss West Germany 900, Grid-Quantifoil Cu Holy Carbon) images of the twelve functionalized silver nanoparticles (images outlined in red indicate additional nanoparticle images on lower magnification). (**a**) 2-aminobenzothiazole-AgNPs (**1-np**) (250,000×), (**b**) 5-methyl-2-aminobenzothiazole-AgNPs (**2-np**) (185,000×), (**c**) 5,6 dimethyl-2-aminobenzothiazole-AgNPs (**3-np**) (250,000×), (**d**) 2-amino-4-chlorobenzothiazole-AgNPs (**4-np**) (185,000×), (**e**) 2-amino-6-bromobenzothiazole-AgNPs (**5-np**) (140,000×), (**f**) 2-methyl-4*H*-3,1-benzoxazin-4-one-AgNPs (**6-np**) (250,000×), (**g**) 2-phenyl-4*H*-3,1-benzoxazin-4-one-AgNPs (**7-np**) (250,000×), (**h**) 3-amino-2-methyl-4(3*H*)-quinazolinone-AgNPs (**8-np**) (250,000×), (**i**) 3-amino-2-phenyl-4(3*H*)-quinazolinone AgNPs (**9-np**) (185,000×), (**j**) 2-butyne-1,4-diol-AgNPs (**10-np**) (250,000×), (**k**) 3-butyn-1-ol-AgNPs (**11-np**) (250,000×), (**l**) heptane-1,7-dioic-AgNPs (**12-np**) (250,000×).

The 12 synthesized silver nanoparticles were further analyzed with TEM. Results showed the formation of nanoparticles with a wide distribution of shapes and sizes, shown in Figure 6. Among the 12 AgNPs, eight were spherically shaped (**1-np**, **3-np**, **5-np**, **6-np**, **7-np**, **9-np**, **11-np**, and **12-np**) whereas the other four displayed nanotube morphologies (**2-np**, **4-np**, **8-np**, and **10-np**). Agglomeration in some of the samples (namely **4-np**, **10-np**, **11-np**, and **12-np**) occurred due to covalent and metallic bond formation as a result of functionalization by the organic ligand.

**Figure 7:**
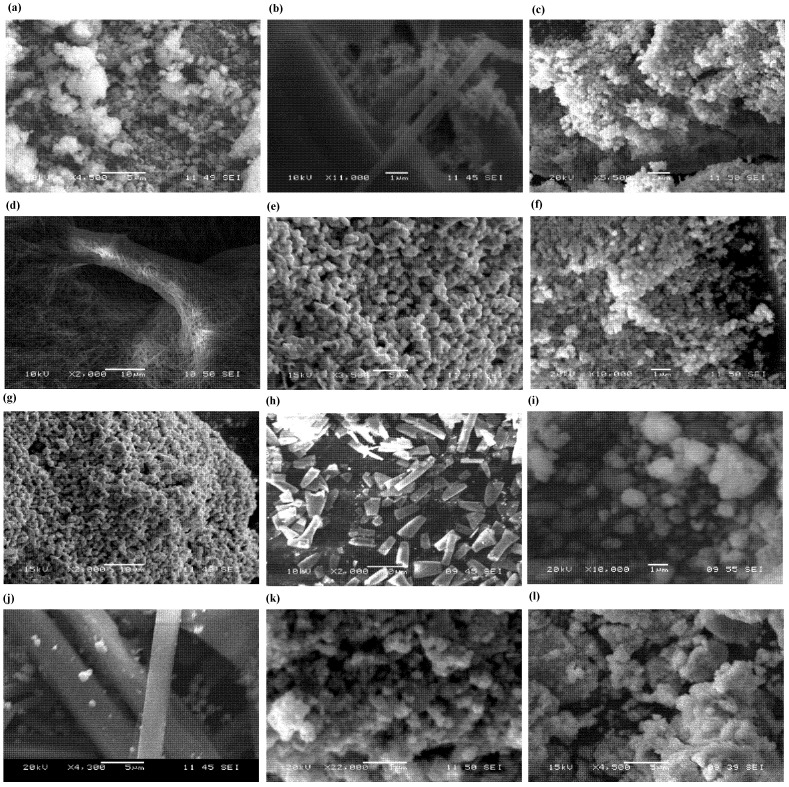
SEM images of the twelve functionalized silver nanoparticles. (**a**) **1-np** (4500×), (**b**) **2-np** (11,000×), (**c**) **3-np** (5500×), (**d**) **4-np** (2000×), (**e**) **5-np** (3500×), (**f**) **6-np** (10,000×), (**g**) **7-np** (2000×), (**h**) **8-np** (2000×), (**i**) **9-np** (10,000×), (**j**) **10-np** (4300×), (**k**) **11-np** (22,000×), (**l**) **12-np** (4500×).

SEM images of the 12 AgNPs were also obtained, yielding the morphological data presented in Figure 7. The morphological details provided by the SEM analyses matched the data shown in the TEM images. Both sets of images confirm spherical morphologies for eight nanoparticles and nanotube morphologies for four nanoparticles. The nanotube-like structure of these four AgNPs could be attributed to high-speed crystal growth or overgrowth within a short time.

### 2.5. Antibacterial Assay Analysis

**Figure 8:**
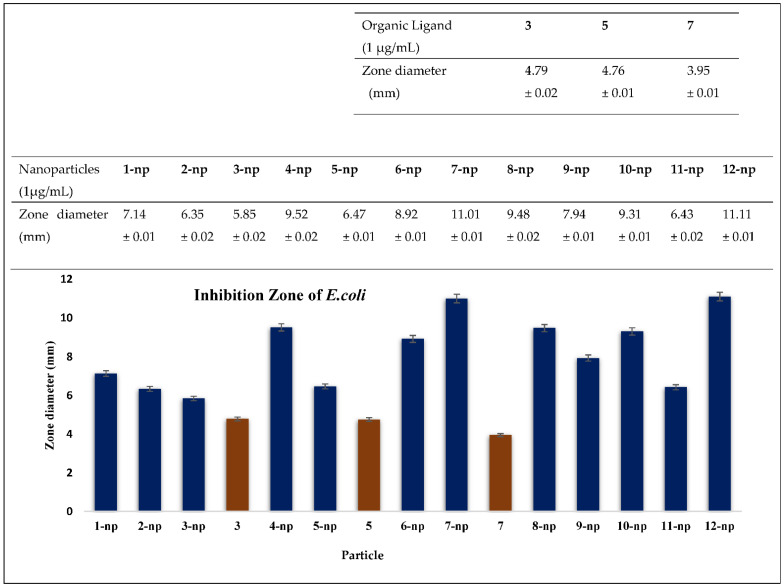
Representative zones of inhibition of the AgNPs with their respective organic ligands (bacterial assays were performed as triplicates and mean values are presented with ± standard errors).

The antimicrobial activities of the 12 conjugated silver nanoparticles and organic compounds **1**–**10** were compared to silver nitrate in a zone of inhibition assay using *E. coli*, a Gram-negative bacterium that has been extensively used in biotechnology, microbiology, and molecular biology [15]. The organism is common, genetically versatile, and can replicate rapidly, making it an excellent choice for this study. Strong antimicrobial activity was observed for silver nitrate (AgNO_3_), with no visual colony formation. Additionally, a white precipitate was observed on the plate. Similarly strong antibacterial activity was apparent for the following nanoparticles with no visible colony formation in the zones of inhibition: **4-np**, **6-np**, **7-np**, **8-np**, **9-np**, **10-np**, and **12-np**. These nanoparticles all had zone of inhibition diameters of approximately 9.00 mm or higher except **9-np**. **7-np** and **12-np** both produced zones of inhibition with diameters higher than 11.00 mm. These two AgNPs show the most promise for further development as antimicrobial agents. Several of the other nanoparticles showed modest activity, including **1-np**, **2-np**, **3-np**, **5-np**, and **11-np**. These organic ligand-functionalized AgNPs all produced zone-of-inhibition diameters of approximately 6.00 mm or above, which is larger than the zones of inhibition produced by any of the singular organic ligands (Figure 8).

Only three of the unconjugated organic compounds showed noticeable antimicrobial activities with full bacterial clearance being the standard for measurable activity. However, following conjugation, all 12 of the organic ligand-functionalized AgNPs demonstrated bacterial clearance to an extent significantly higher than their respective singular ligands [16,17].

## 3. Discussion

The zone-of-inhibition assay demonstrated strong antimicrobial activities in **4-np**, **6-np**, **7-np**, **8-np**, **9-np**, **10-np**, and **12-np**. Neither strong nor significant antibacterial activity was found in the organic ligands corresponding to the AgNPs. This signifies that the conjugation of the silver nanoparticles to the organic ligands was the factor increasing antimicrobial activity. Among these seven conjugated AgNPs, three had nanotube-like morphologies (**4-np**, **8-np**, and **10-np**) and four had spherical morphologies (**6-np**, **7-np**, **9-np**, and **12-np**). These data alone show no correlation between conjugated AgNP morphology and antimicrobial ability. However, in combination with size data, the shapes of the AgNPs gain significance. The three nanotube-shaped AgNPs were additionally three of the largest particles analyzed with sizes of 278.25 nm, 152.42 nm, and 218.60 nm. The **2-np** particle, which was also nanotube-shaped, showed significant but not strong antibacterial activity, with a zone-of-inhibition diameter of 6.35 mm; **2-np** only had a size of 121.70 nm, which is lower than those of the other nanotube-shaped AgNPs. This difference in both size and antimicrobial activity could be an indicator that larger nanotube-shaped AgNPs tend to be more effective for antibacterial purposes. As for **5-np**, it was also relatively large, 171.25 nm, but was spherical. Antimicrobial activity was determined to be significant, but not strong like the other large particles. This was concluded from the 6.47 mm clearance diameter. The lowered antibacterial capabilities of **5-np** show that the size benefit for bacterial inhibition is most likely exclusive to nanoparticles with nanotube-like morphologies.

In contrast to this, the opposite seems to hold true for the spherically shaped nanoparticles, but with less strong of a correlation. Both **7-np** and **9-np** have the smallest sizes of all 12 nanoparticles: 25.02 nm and 38.72 nm, respectively. The high antibacterial activity for particles **7-np** and **9-np** could be due to the small sizes and spherical shapes providing a more facile transport through the *E. coli*’s cytoplasmic membrane, increasing biodistribution [18]. Particles **6-np** and **12-np**, however, are much more average in size: 86.02 nm to 76.60 nm, respectively. This signifies that antimicrobial capabilities are influenced by factors other than size and shape.

A few of these factors to be examined include agglomeration, organic ligand identity, and organic ligand functionalized AgNP complexity. All nanoparticles for which agglomeration was observed (**4-np**, **10-np**, **11-np**, and **12-np**) except for **11-np** showed strong antimicrobial activity with zones of inhibition above 9.00 mm. While the exception of **11-np** lessens the validity of this correlation, links between agglomeration and antimicrobial activity could yield useful results with larger samples. The wide distribution between both the properties of the varying organic ligands and their respective antimicrobial activities when conjugated with AgNPs also suggests that the individual identities of the ligands greatly affect results. Both AgNPs with benzoxazine (**6-np** and **7-np**) performed exceptionally well in the zone-of-inhibition assay, and the same held true for the two AgNPs with quinazolinone (**8-np** and **9-np**). It is notable that the use of quinazolinone derivatives for conjugation with AgNPs for antimicrobial activity has been explored and found to be effective [8]. However, this pairing pattern was not matched by the other nanoparticles, showing that the actual changes made to the compounds from which the organic ligands were derived influenced antimicrobial activity as well. Furthermore, functionalization of the nanoparticles themselves was shown to alter antibacterial ability. The organic ligands **3**, **5**, and **7** showed promising antimicrobial activity in the zone-of-inhibition assay, with moderate bacterial clearances. Despite this, when conjugated with nanoparticles, only **7-np** showed strong antibacterial activity. These results exemplify the structural alteration caused by the conjugation of the AgNPs. This outcome confirms that not only the structure of the organic ligand but the structural interactions between the ligand and the silver must be considered in the synthesis of new organic ligand functionalized AgNP-based antimicrobial agents.

The results presented here suggest a high potential for the discovery of new antimicrobial agents using a variety of organic ligands. Studies conducted with a larger set of organic ligand-functionalized AgNPs could provide further insight into the conceptual link between organic ligand differentiation and antimicrobial activity distribution. Additional biological analysis could also be useful. This study presents numerous correlations between the characteristics of organic ligand-conjugated AgNPs and their respective antibacterial capabilities. However, more research is needed to confirm definitive causation for the proposed factors. The variety of organic ligands used led to a differentiation between the abilities of the conjugated nanoparticles, resulting in seven strong candidates for use as antimicrobial agents.

## 4. Materials and Methods

**Scheme 1:**
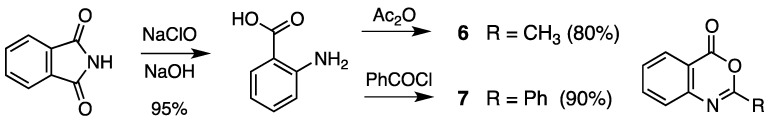
Reaction scheme for the synthesis of the benzoxazine-4-one-derivatives.

**Scheme 2:**
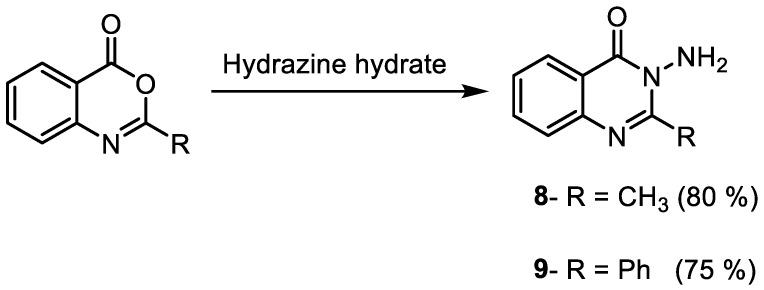
Reaction scheme for the synthesis of the quinazolinone derivatives.

The molecular structures of the twelve organic ligands used for this study are represented in Figure 1. The 2-aminobenzothiazole derivatives (**1**–**5**) were obtained from Fisher Scientific (Waltham, MA, USA) and the two benzoxazine derivatives, 2-methyl-4*H*-3,1-benzoxazin-4-one (**6**) and 2-phenyl-4*H*-3,1-benzoxazin-4-one (**7**) were synthesized according to the procedures described below in Scheme 1. The other two quinazolinone derivatives, 3-amino-2-methyl-4(3*H*)-quinazolinone (**8**) and 3-amino-2-phenyl-4(3*H*)-quinazolinone (**9**), were synthesized from ligands **6** and **7** respectively, as illustrated in Scheme 2. Silver nitrate was used as the starting material for the synthesis of all twelve silver nanoparticles, and all reagents and chemicals were obtained from Fisher Scientific (Waltham, MA, USA). The successful synthesis of all derivatives was confirmed using proton nuclear magnetic resonance (^1^H NMR), carbon nuclear magnetic resonance (C-13 NMR), and liquid chromatography–mass spectrometry (LC-MS).

### 4.1. Synthesis of Benzoxazin-4-One Derivatives

**2-aminobenzoic acid:** Sodium hydroxide (10.00 g, 250 mmol) was added to a 250 mL Erlenmeyer flask containing ice-cold water (60.00 mL) and a magnetic stir bar. The solution was stirred until the sodium hydroxide dissolved. The phthalimide (10.00 g, 73 mmol) was quickly added to the sodium hydroxide solution. An ice bath was placed around the flask and stirring was continued. Sodium hypochlorite (14.00 mL, 5.00 M) was added to the solution, which was stirred for another 15 min before removal from the ice bath. The solution, which had turned a faint-yellow color, was then heated to 75 °C. This temperature was maintained for an additional 15 min. The solution was cooled in an ice bath and 10.00 mL of the solution was transferred to a small beaker. Hydrochloric acid (8.00 M) was added until the solution reached a neutral pH (7.0). Then, glacial acetic acid (10.00 mL) was added, and the resulting precipitate was washed and recrystallized with cold water.

**(6) 2-methyl-4*H*-3,1-benzoxazin-4-one**: A mixture of anthranilic acid (10.00 mmol) and acetic anhydride (1.50 mL) was heated at 150 °C for 2.5 h. Excess acetic anhydride was then removed under reduced pressure and the resulting solid was triturated with petroleum ether, collected by filtration, and dried in a vacuum.

**(7) 2-phenyl-4*H*-3,1-benzoxazin-4-one**: Anthranilic acid (5.00 g, 22 mmol) was dissolved slowly at room temperature in 10.00 mL of anhydrous pyridine with continuous stirring. The solution was heated by the addition of anhydrous pyridine, then cooled to 10 °C in a water bath. Cooling of the mixture contributed to the production of solid crystals. Benzoyl chloride (2.60 mL, 22 mmol) was then slowly added to 10.00 mL of anhydrous pyridine and stirred for 30 min. The resulting solid was washed with water and treated with aqueous sodium bicarbonate to remove any unreacted acid. The reaction mixture was left stirring overnight when the resulting product was not formed immediately. The reaction can be slowed by various conditions, but the crude can be recrystallized using ethanol in these situations. Following the addition of the DI water and the dissolution of the precipitate in its entirety, the final solid product was formed through the neutralization of the reaction mixture using sodium bicarbonate.

### 4.2. Synthesis of Quinazolinone Derivatives

**(8) 3-amino-2-methyl-4(3*H*)-quinazolinone**: Hydrazine hydrate (10.00 mL, 99%) was added to a solution containing 2-methyl-4*H*-3,1-benzoxazin-4-one (**6**) (3.00 g, 20 mmol) and absolute ethanol (15.00 mL). The mixture was refluxed for 27 h, cooled, and the resulting precipitate was filtered and recrystallized from water.

**(9) 3-amino-2-phenyl-4(3*H*)-quinazolinone**: A mixture of 2-phenyl-4*H*-1,3-benzoxazin-4-one (**7**) (100 mg, 0.45 mmol) and hydrazine hydrate (4.0 mL, 82 mmol) was refluxed for 25 min at 200–250 °C using an oil bath. The mixture was then cooled to room temperature and poured over ice for the collection of the precipitate. The resulting solid was then separated and purified using ethanol [17].

### 4.3. Synthesis of Silver Nanoparticles

For the syntheses of the AgNPs, the benzothiazole, benzoxazine, quinazolinone, heptane-1,7-dioic, 2-butyne-1,4-diol, and 3-butyn-1-ol derivatives were conjugated with silver to generate the twelve functionalized silver nanoparticles. Each organic ligand derivative (**1**–**12**) (10.00 mg) was reacted aqueously with silver nitrate in ethanol (0.1 mM, 3.00 mL). The mixture was then stirred for 6 h in the dark and a solution of NaBH_4_ (10 µL, 6 mM) was added. The mixture transitioned from transparent to light brown following the addition of the reducing agent, suggesting the reduction of silver ions and the formation of silver-functionalized nanoparticles. All organic derivatives (**1**–**12**) were conjugated through adjustments to the diverse volume ratio (*v*/*v*) of the twelve derivatives to the silver nitrate solution [14]. The procedure was performed in duplicate under red light at 620 nm for 6 h and the nanoparticle formation yields were compared.

### 4.4. Antibacterial Assay

The effects of the synthesized nanoparticles on bacterial growth were evaluated using a halo assay. A bacterial lawn was generated from a saturated overnight culture of *E. coli* bacteria (DH5ɑ) grown in lysogeny broth (LB), diluted 100-fold in LB, and spread onto LB agar plates using sterile glass beads. Plates were dried for approximately 15 min before solutions of the organic ligand-modified AgNPs (1 µg/mL, 3 µL) were spotted onto the plates. As a control, the organic ligands corresponding to their respective nanoparticles were also spotted on the plates (1 µg/mL, 3 µL). Ampicillin (100 mg/mL, 3 µL, Fisher Bioreagents) was used as a positive control and ultrapure water (3 µL) was used as a negative control. An AgNO_3_ solution was included as a reference. The plates were then incubated at 37 °C overnight before imaging with a BioRad ChemiDoc MP Imaging System and Image Lab Software 5.2 (Bio-Rad, Hercules, CA, USA). Analysis was performed using Mac OS 10.15.7 Preview, Microsoft PowerPoint, and NIH ImageJ. Antimicrobial activity was determined by the clearance of bacteria. Each assay was performed using three independent biological replicates.

## 5. Conclusions

Twelve silver nanoparticles functionalized with organic ligands were synthesized with red light and characterized through a variety of methods. Various analyses confirmed successful nanoparticle conjugation with the organic ligands. A variety of nanoparticle sizes and shapes also resulted from the array of organic ligands used, and all tested AgNPs exhibited some antibacterial capabilities against *E. coli*. However, seven of the AgNPs showed especially strong antimicrobial properties, indicating the potential for further development. A correlation between the morphological details of the conjugated nanoparticles and the extent of their antimicrobial activities was observed. The differentiation between the organic ligands led to a wide distribution of antibacterial capabilities of the produced AgNPs. While nanoparticles are more effective and less toxic than traditional antibacterial agents, further research on the biological effects of these nanoparticles must be conducted. More research must be conducted on the seven AgNPs possessing strong antibacterial capabilities. Optical density (OD) measurement of the bacterial assay should be analyzed for more precise quantitative data. Future investigation of the nanoparticle solutions at varying concentrations along with the determination of MIC values would also be beneficial. Nevertheless, this research reveals promising new insights into the use of organic ligand-functionalized AgNPs as antimicrobial agents with high potency, reduced toxicity, and strong mechanisms of activity. These particles represent both concrete and conceptual contributions to the field of nanomedical chemistry.

## Figures and Tables

**Table 1:** Average sizes and zeta potentials for the 12 AgNPs.

Nanoparticles	Size (nm)	Zeta Potential (mV)
2-aminobenzothiazole-AgNPs (**1-np**)	87.30	−42.3
5-methyl-2-aminobenzothiazole-AgNPs (**2-np**)	121.70	−42.7
5,6-dimethyl-2-aminobenzothiazole-AgNPs (**3-np**)	56.54	−40.4
2-amino-4-chlorobenzothiazole-AgNPs (**4-np**)	278.25	−38.5
2-amino-6-bromobenzothiazole-AgNPs (**5-np**)	171.25	−37.7
2-methyl-4*H*-3,1-benzoxazine-4-one-AgNPs (**6-np**)	86.02	−56.3
2-phenyl-4*H*-3,1-benzoxazin-4-one-AgNPs (**7-np**)	25.02	−59.2
3-amino-2-methyl-4(3*H*)-quinazolinone-AgNPs (**8-np**)	152.42	−50.8
3-amino-2-phenyl-4(3*H*)-quinazolinone-AgNPs (**9-np**)	38.72	−53.2
2-butyne-1,4-diol-AgNPs (**10-np**)	218.60	−21.4
3-butyn-1-ol-AgNPs (**11-np**)	58.60	−19.4
heptane-1,7-dioic-AgNPs (**12-np**)	76.60	−16.3

## Data Availability

Not applicable.

## Supplementary Materials

The following supporting information can be downloaded at https://www.mdpi.com/article/10.3390/ijms232315251/s1.

## Abbreviations

AgNPs	silver nanoparticles
NPs	nanoparticles
NaBH_4_	sodium borohydride
h	hour(s)
min	minute(s)
H_2_O	Water
