# Supplementary material for: Synthesis and Characterization of Ligand-Stabilized Silver Nanoparticles and Comparative Antibacterial Activity against E. coli
Source: Int J Mol Sci. 2022 Dec 3;23(23):15251. doi: 10.3390/ijms232315251 (PMC9740489; doi:10.3390/ijms232315251)
Supplement: Supplementary file 1 [file ijms-23-15251-s001.zip › ijms-1956271-supplementary.pdf]

Supporting Information

Table of Contents

Synthesis Procedure of Benzoxazine Derivatives S2
Synthesis Procedure of Quinazolinone Derivatives S4
NMR Data of the Compounds S5
LCMS Data of the Compounds S13
Comparison of UV-Vis Spectra of AgNPs with Organic Ligands S15
FTIR Analysis of AgNPs with Organic Ligands S17
EDAX Analysis of AgNPs S19
Size Distribution Histogram of AgNPs S21
Zeta Potential Analysis of AgNPs S23
Antibacterial Assay S25

Synthesis Procedure of Benzoxazine Derivatives

(A) 2-aminobenzoic acid: Sodium hydroxide (10.00 g, 250 mmol) was added to a 250 mL Erlenmeyer flask containing ice-cold water (60.00 mL) and a magnetic stir bar. The solution was stirred until the sodium hydroxide dissolved and the phthalimide (10.00 g, 73 mmol) was quickly added to the sodium hydroxide solution. An ice bath was placed around the flask as stirring was continued. Sodium hypochlorite (14.00 mL, 5.00 M) was added to the solution, which was stirred for another 15 min before removal from the ice bath. The solution, which had turned a faint yellow color, was then heated to 75 ˚C. This temperature was maintained for an additional 15 min. The solution was then cooled in an ice bath and 10.00 mL was transferred to a small beaker. Hydrochloric acid (8.00 M) was then added until the solution reached a neutral pH (7.0). Then, glacial acetic acid (10.00 mL) was added, and the resulting precipitate was washed and recrystallized with cold water.

(B) 2-methyl-4*H*-3,1-benzoxazin-4-one (6): A mixture of anthranilic acid (10.00 mmol) and acetic anhydride (1.50 mL) was heated at 150 ˚C for 2.5 h. Excess acetic anhydride was then removed under reduced pressure and the resulting solid was triturated with petroleum ether, collected by filtration, and dried in a vacuum (Yield: 80%). ^{1}H NMR (400 MHz, DMSO) δ 7.25 (d, J = 7.7 Hz, 1H), 7.06 (t, J = 7.7 Hz, 1H), 6.75 (d, J = 7.7 Hz, 1H), 6.74 – 6.70 (t, 1H), 1.66 (s, 3H). ^{13}C NMR (101 MHz, $CDCl_3$) δ 160.27, 146.43, 136.53, 128.46, 128.19, 126.38, 120.41, 116.67, 21.33. MS (LCMS-ESI) m/z $[M+H]^+$ cacld for $C_9H_7NO_2{}^+$ 162.1, found 162.1.

(C) 2-phenyl-4*H*-3,1-benzoxazin-4-one (7): Anthranilic acid (5.00 g, 22 mmol) was dissolved slowly at room temperature in 10.00 mL of anhydrous pyridine with continuous stirring. The solution was heated by the addition of anhydrous pyridine, then cooled to 10 ˚C in a water bath. Cooling of the mixture contributed to the production of solid crystals. Benzoyl chloride (2.60 mL, 22 mmol) was then slowly added to 10.00 mL of anhydrous pyridine and stirred for 30 min. The resulting solid was washed with water and treated with aqueous sodium bicarbonate to remove any unreacted acid. The reaction mixture was left stirring overnight when the resulting product was not formed immediately. The reaction can be slowed by various conditions and the crude can be recrystallized using ethanol in this situation. Following the addition of the DI-water and the dissolution of all the precipitate, the final solid product was formed through the neutralization of the reaction mixture using sodium bicarbonate (Yield: 90%) white solid. (Needle-shaped) IR values (cm^{-1}) are as indicated: C=O-1780-1650, C=N-1650-1550. ^{1}H NMR (400 MHz, $CDCl_3$) δ 8.33 (d, *J* = 7.3 Hz, 2H), 8.26 (d, *J* = 7.8 Hz, 1H), 7.84 (t, *J* = 7.1 Hz, 1H), 7.71 (d, *J* = 8.0 Hz, 1H), 7.59 (t, *J* = 7.3 Hz, 1H), 7.56 – 7.45 (m, 3H).^{13}C NMR (101 MHz, $CDCl_3$) δ 159.57, 157.17, 147.03, 136.55, 132.61, 130.57, 130.29, 128.74, 128.61, 128.33, 127.24, 117.06. MS (LCMS-ESI) m/z $[M+H]^+$ cacld for $C_{14}H_9NO_2{}^+$ 224.2, found 224.1.

Synthesis Procedure of Quinazolinone Derivatives

(D) 3-amino-2-methyl-4(3*H*)-quinazolinone (8): Hydrazine hydrate (10.00 mL, 99%) was added to a solution containing 2-methyl-4*H*-3,1-benzoxazin-4-one (**6**) (3.00 g, 20 mmol) and absolute ethanol (15.00 mL). The mixture was refluxed for 27 h, cooled, and the resulting precipitate was filtered and recrystallized from water (Yield: 80%). ^{1}H NMR (400 MHz, DMSO) δ 8.10 (d, *J* = 7.1 Hz, 1H), 7.84 – 7.70 (t, 1H), 7.60 (d, *J* = 8.1 Hz, 1H), 7.48 (t, *J* = 7.5 Hz, 1H), 2.58 (s, 3H). ^{13}C NMR (101 MHz, DMSO) δ 160.02, 155.50, 146.59, 133.90, 126.60, 125.91, 125.84, 119.73, 21.85. MS (LCMS-ESI) m/z $[M+H]^+$ cacld for $C_9H_9N_3O^+$ 176.1, found 176.1.

(E) 3-amino-2-phenyl-4(3*H*)-quinazolinone (9): A mixture of 2-phenyl-4*H*-1,3-benzoxazin-4-one (**7**) (100 mg, 0.45 mmol) and hydrazine hydrate (4.0 mL, 82 mmol) was refluxed for 25 min at 200-250 ˚C using an oil bath. The mixture was then cooled to room temperature and poured over ice for the collection of the precipitate. The resulting solid was then separated and purified using ethanol (Yield: 75%). ^{1}H NMR (400 MHz, DMSO) δ 8.20 (d, *J* = 7.8 Hz, 1H), 7.86 (t, *J* = 7.7 Hz, 1H), 7.83 – 7.79 (m, 2H), 7.72 (d, *J* = 8.1 Hz, 1H), 7.58 (t, *J* = 7.5 Hz, 1H), 7.53 – 7.44 (m, 3H).^{13}C NMR (101 MHz, $CDCl_3$) δ 161.56, 154.54, 147.06, 134.42, 134.12, 130.20, 129.25, 128.17, 127.91, 127.00, 126.59, 120.15. MS (LCMS-ESI) m/z $[M+H]^+$ cacld for $C_{14}H_{11}N_3O^+$ 238.2, found 238.1.

NMR Data of the Compounds

^{1}H NMR of 2-methyl-4*H*-3,1-benzoxazin-4-one (6)

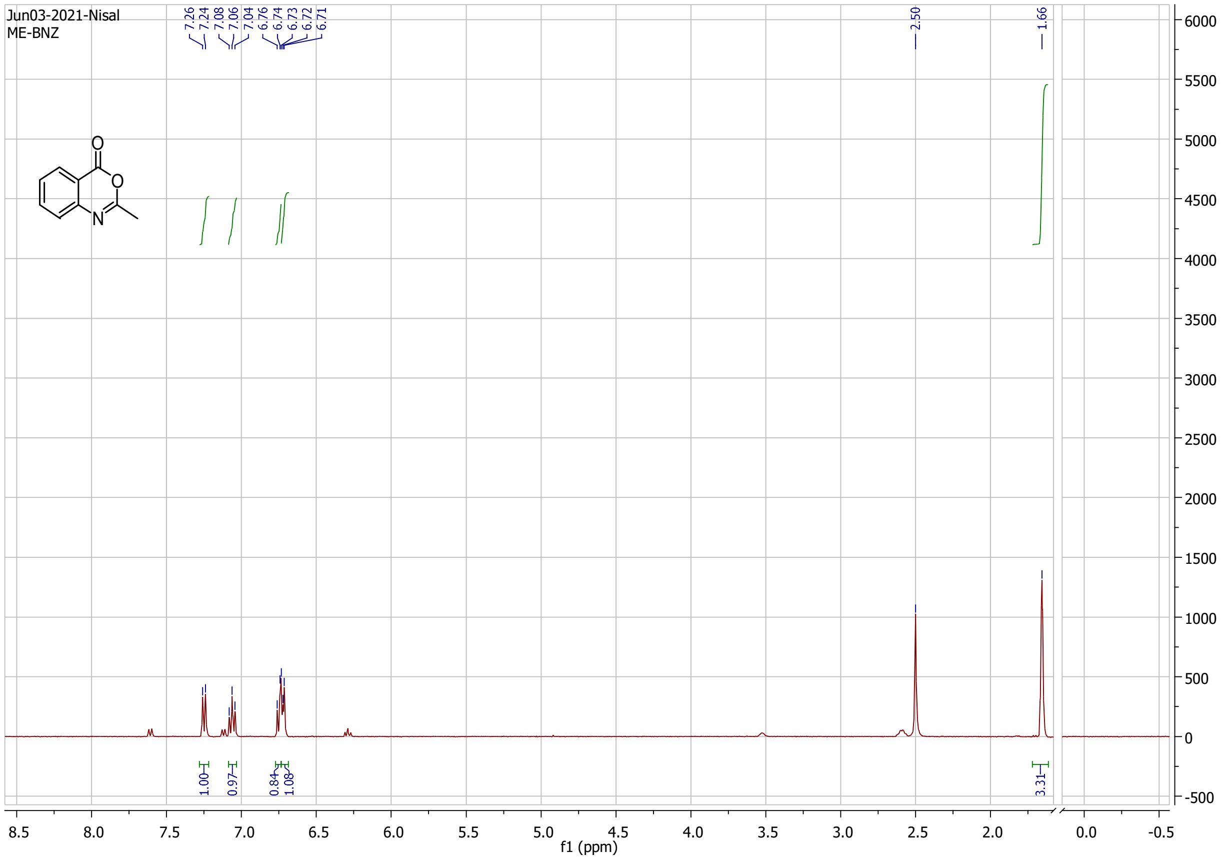

C-13 NMR of 2-methyl-4*H*-3,1-benzoxazin-4-one (6)

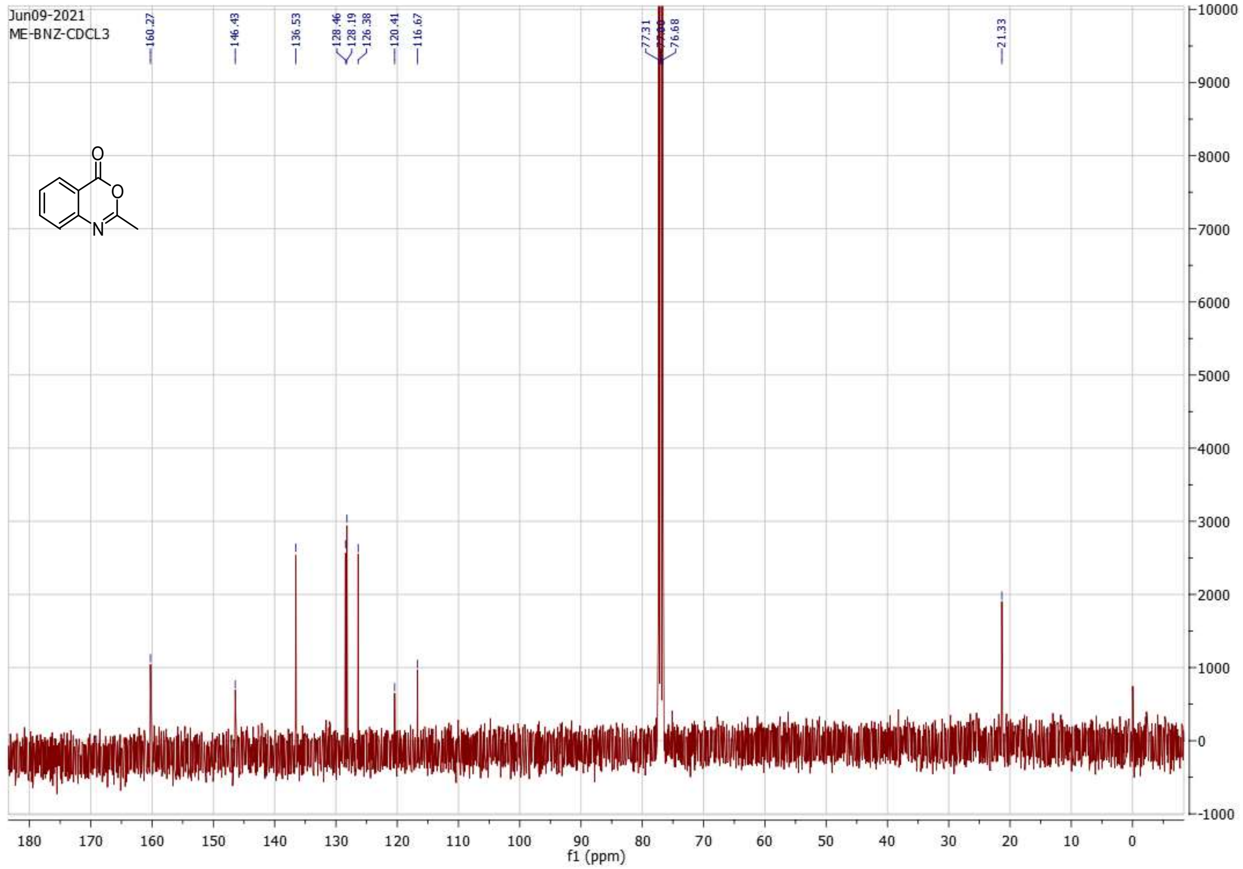

^{1}H NMR of 2-phenyl-4*H*-3,1-benzoxazin-4-one (7)

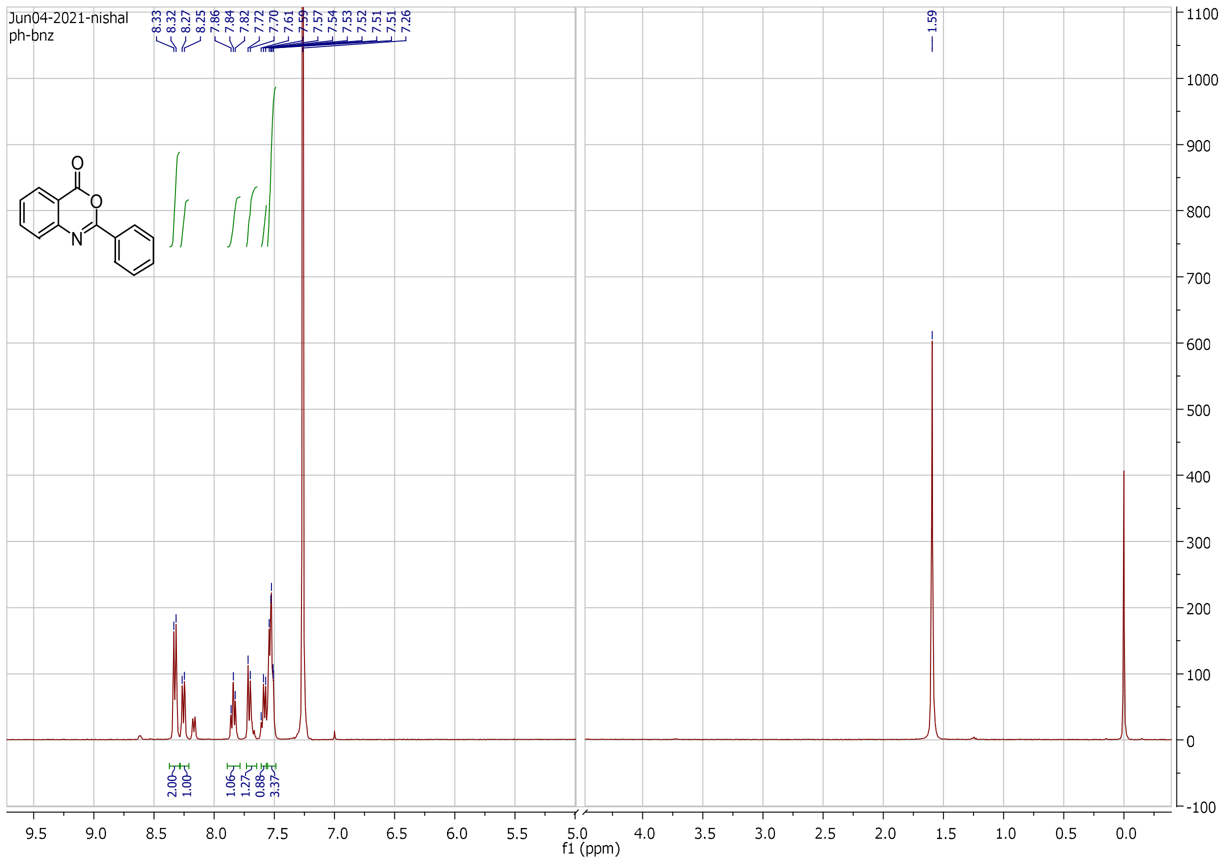

C-13 NMR of 2-phenyl-4*H*-3,1-benzoxazin-4-one (7)

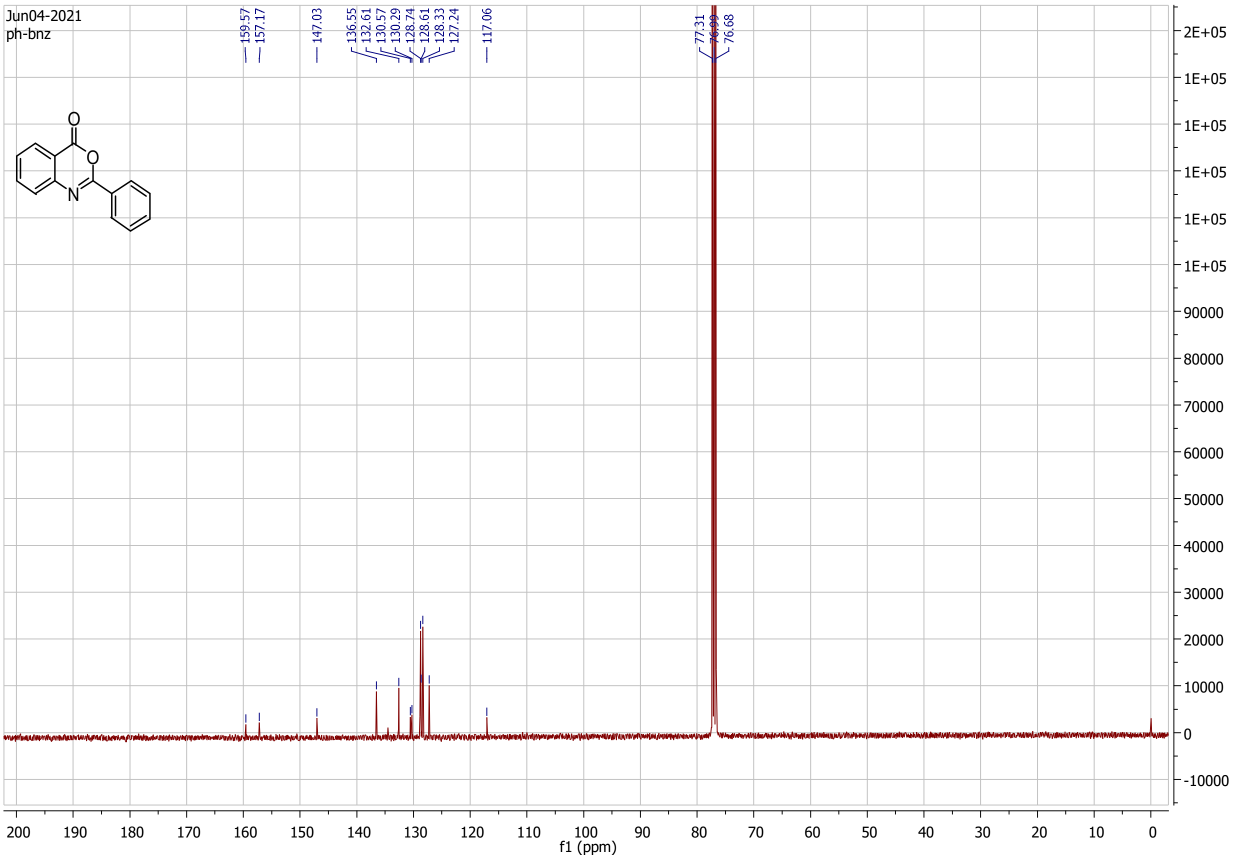

^{1}H NMR of 3-amino-2-methyl-4(3*H*)-quinazolinone (8)

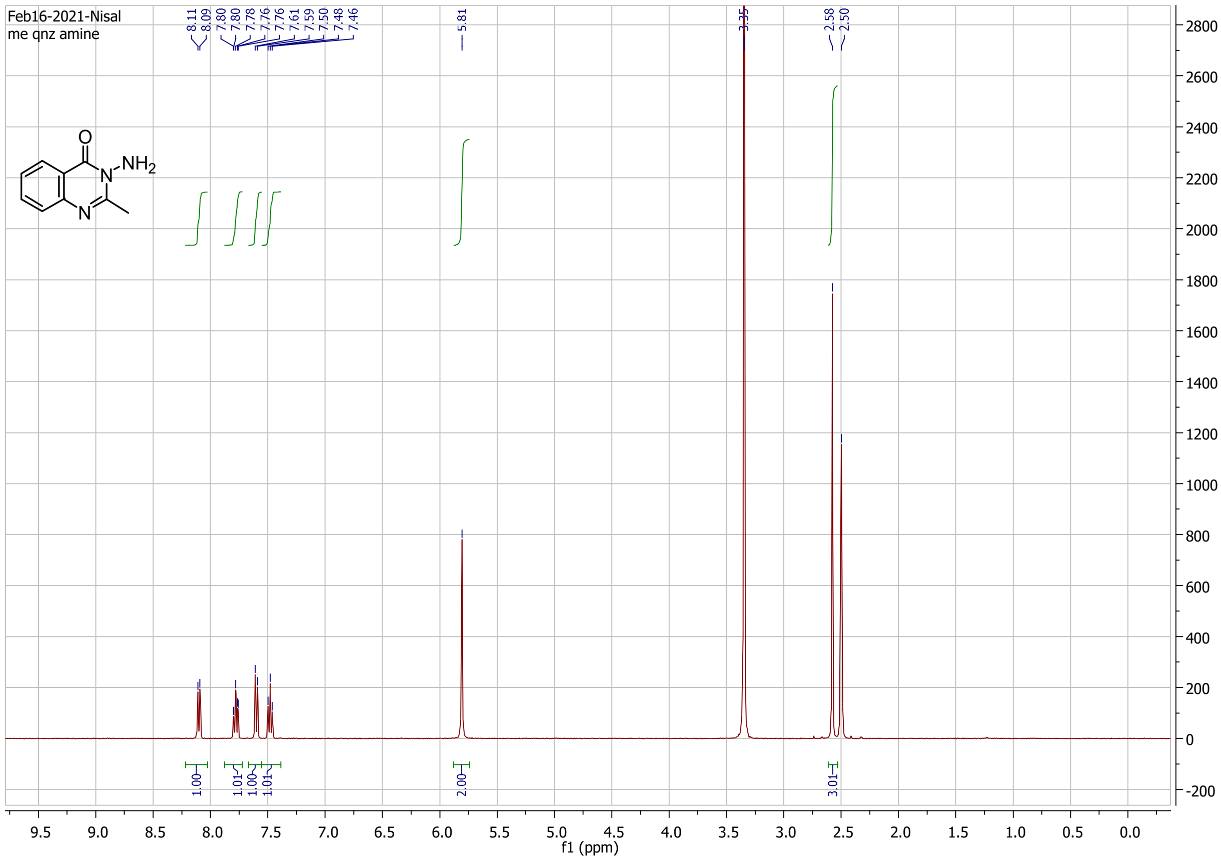

C-13 NMR of 3-amino-2-methyl-4(3*H*)-quinazolinone (8)

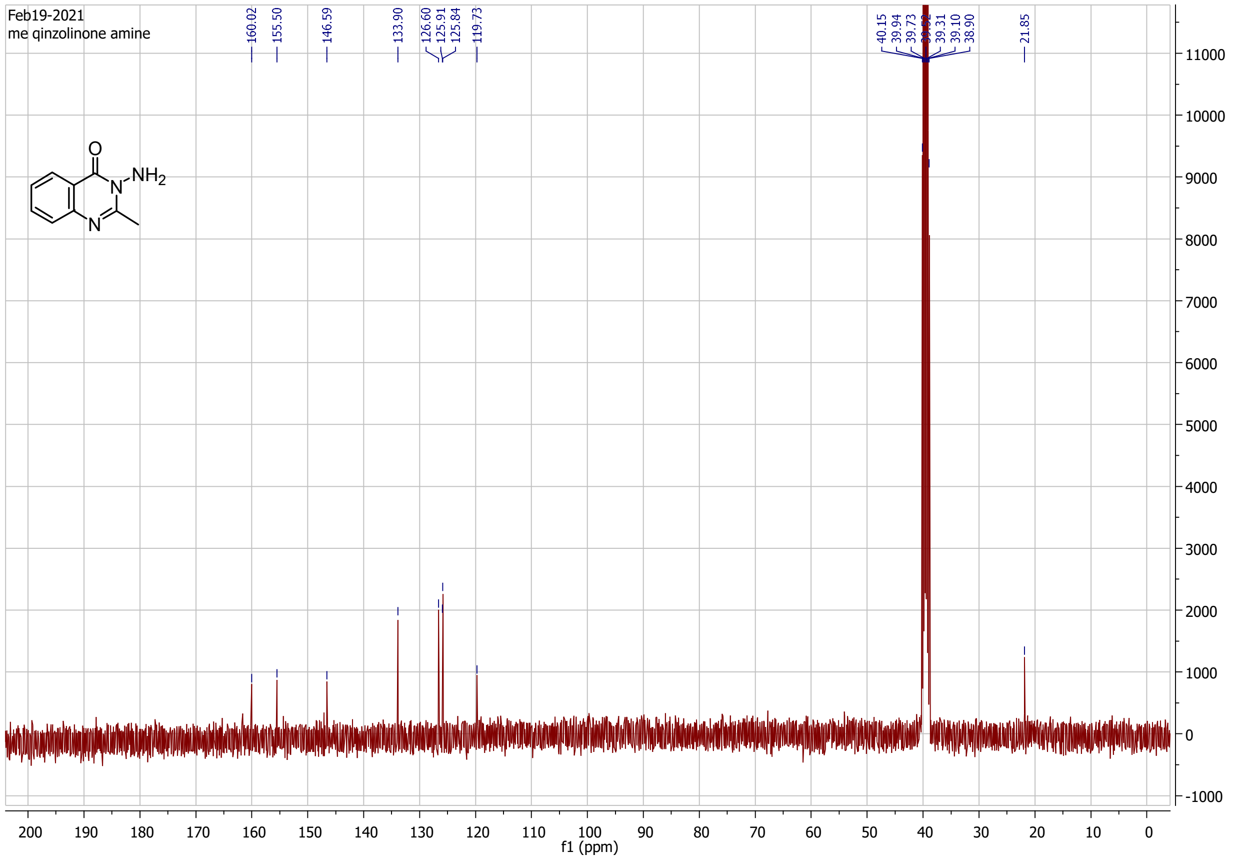

^{1}H NMR of 3-amino-2-phenyl-4(3*H*)-quinazolinone (9)

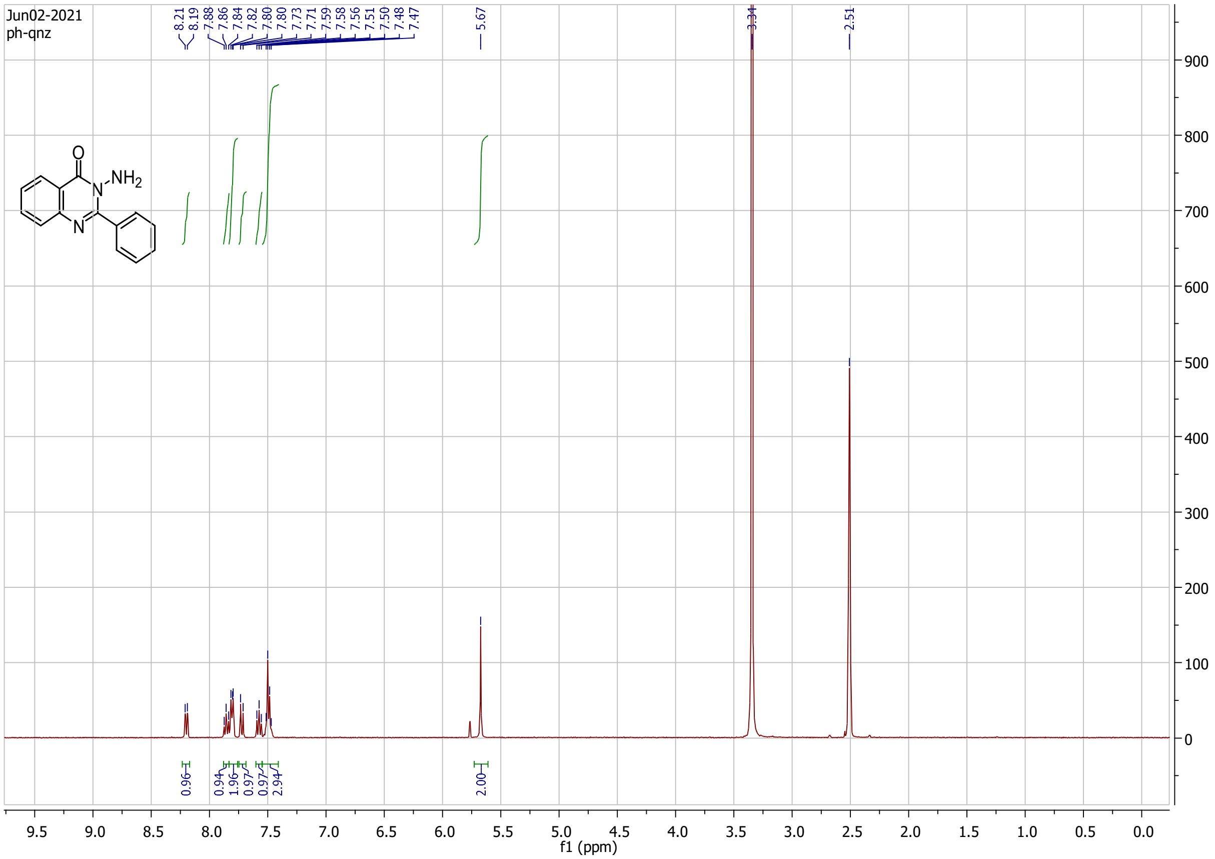

C-13 NMR of 3-amino-2-phenyl-4(3*H*)-quinazolinone (9)

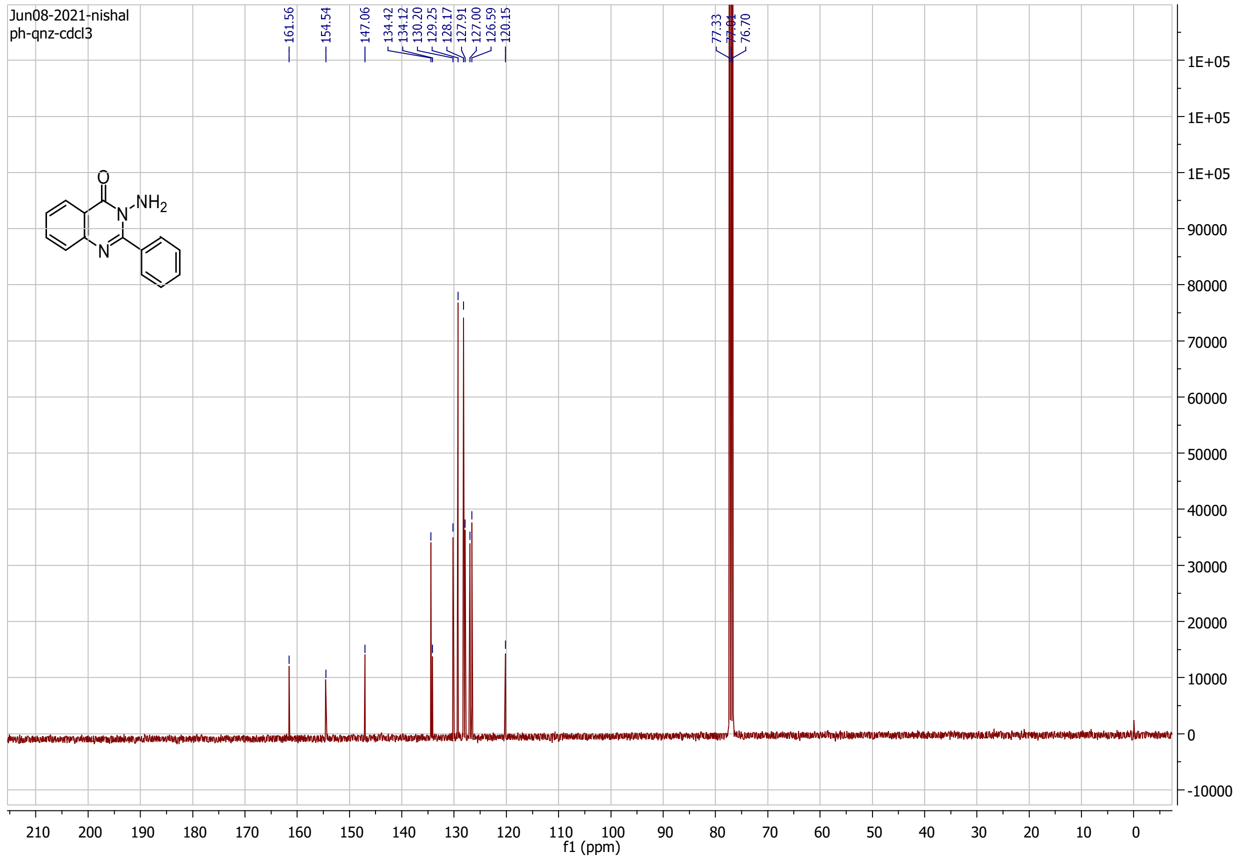

LCMS Data of the Compounds

2-methyl-4*H*-3,1-benzoxazin-4-one (6)

MS (LCMS-ESI) m/z [M+H]$^+$ cacld for $C_9H_7NO_2{}^+$ 162.1, found 162.1

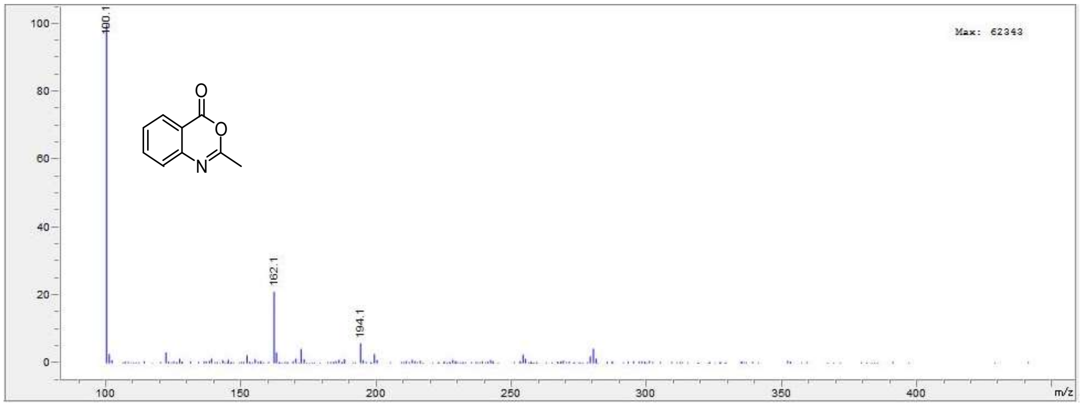

2-phenyl-4*H*-3,1-benzoxazin-4-one (7)

MS (LCMS-ESI) m/z [M+H]$^+$ cacld for $C_{14}H_9NO_2{}^+$ 224.2, found 224.1

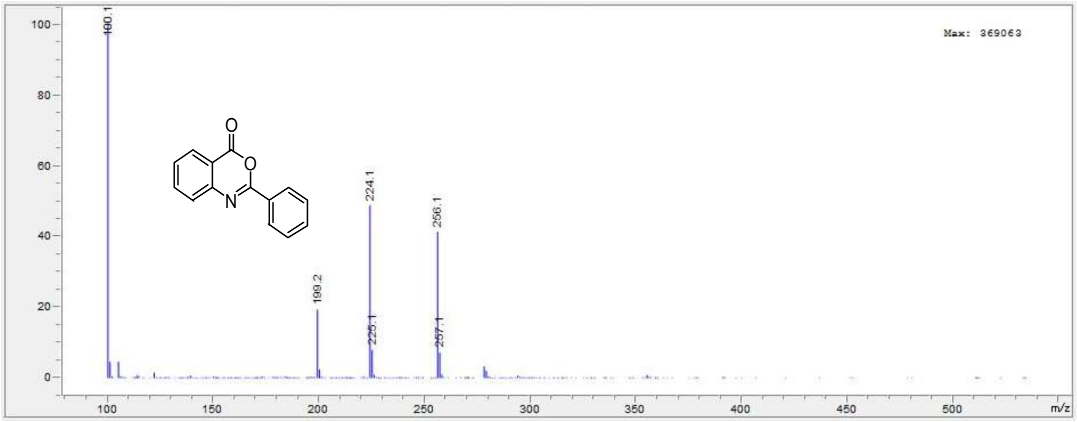

3-amino-2-methyl-4(3*H*)-quinazolinone (8)

MS (LCMS-ESI) m/z $[M+H]^+$ cacld for $C_9H_9N_3O^+$ 176.1, found 176.1

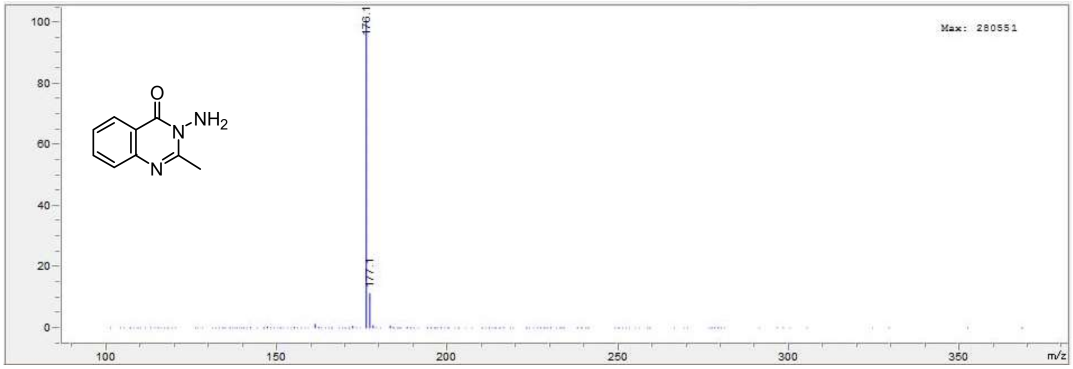

3-amino-2-phenyl-4(3*H*)-quinazolinone (9)

MS (LCMS-ESI) m/z $[M+H]^+$ cacld for $C_{14}H_{11}N_3O^+$ 238.1, found 238.1

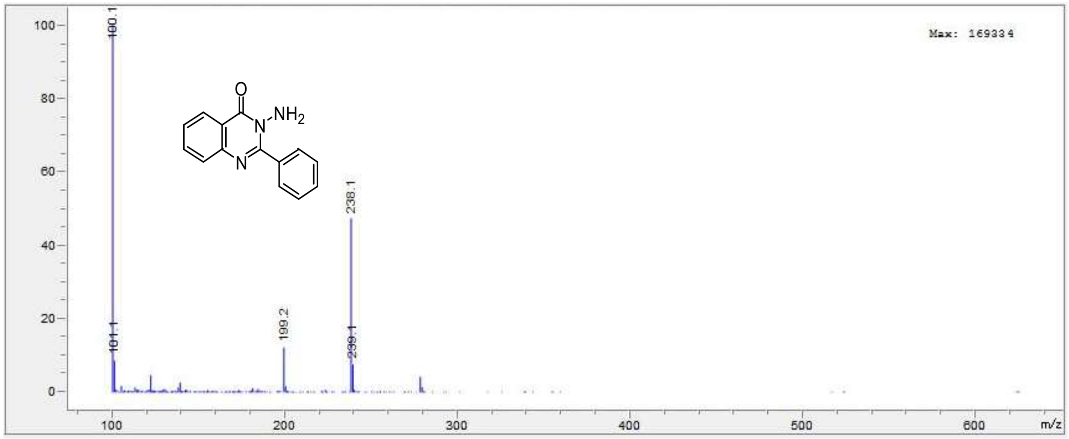

Comparison of UV-Vis Spectra of AgNPs with Organic Ligands

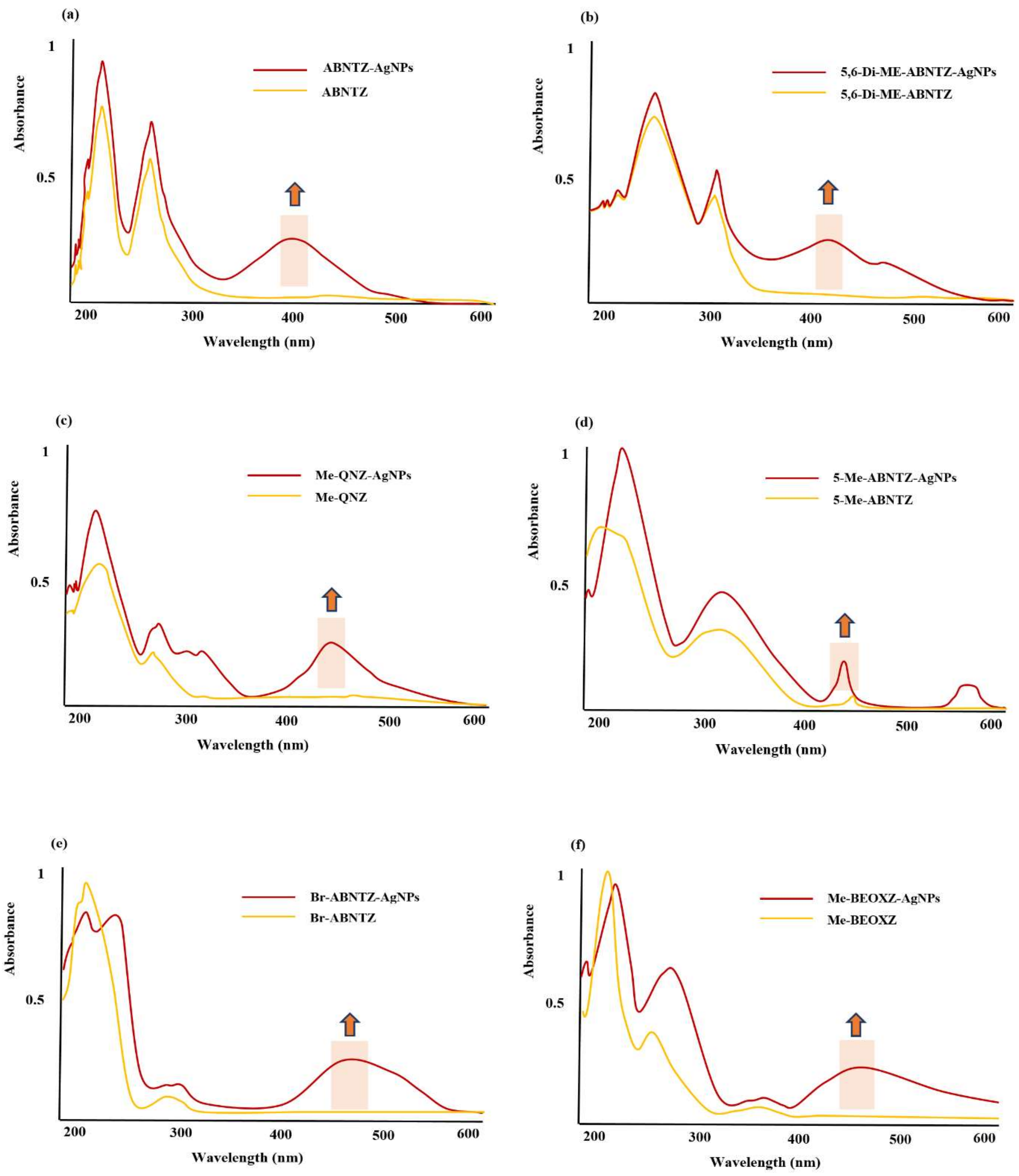

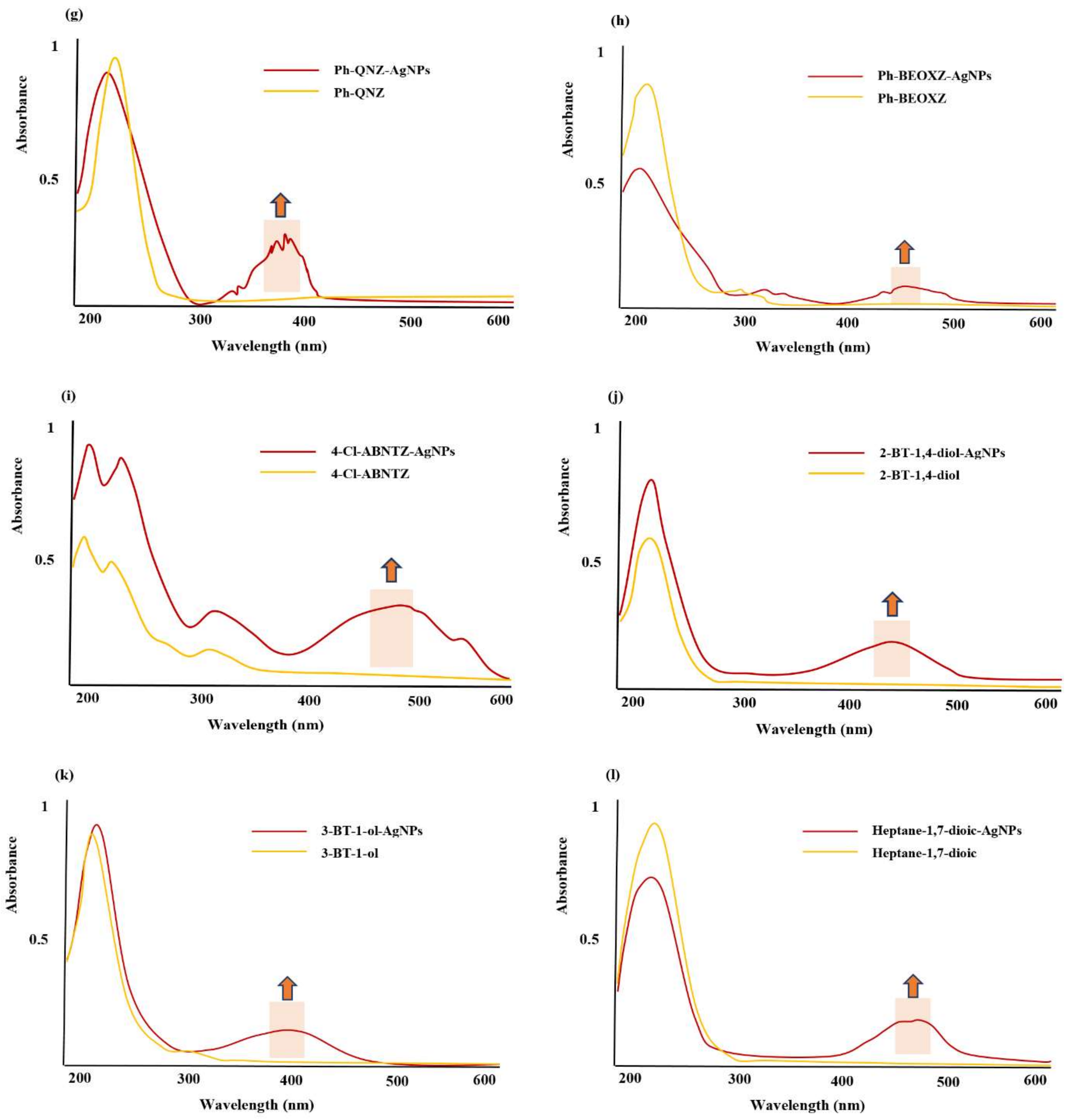

Figure S1. UV spectra analysis of conjugated AgNPs (Red line) with its organic ligand (Yellow line) (a) 2-aminobenzothiazole/2-aminobenzothiazole-AgNPs, (b) 5,6,-dimethyl-2-aminobenzothiazole/5,6,-dimethyl-2-aminobenzothiazole-AgNPs, (c) 3-amino-2-methyl-4(3*H*)-quinazolinone/3-amino-2-methyl-4(3*H*)-quinazolinone-AgNPs, (d) 5-methyl-2-aminobenzothiazole/5-methyl-2-aminobenzothiazole-AgNPs, (e) 2-amino-6-bromobenzothiazole/2-amino-6-bromobenzothiazole-AgNPs, (f) 2-methyl-4*H*-3,1-benzoxazin-4-one/2-methyl-4*H*-3,1-benzoxazin-4-one-AgNPs, (g) 3-amino-2-phenyl-4(3*H*)-quinazolinone/3-amino-2-phenyl-4(3*H*)-quinazolinone-AgNPs, (h) 2-phenyl-4*H*-3,1-benzoxazin-4-one/2-phenyl-4*H*-3,1-benzoxazin-4-one-AgNPs, (i) 2-amino-4-chlorobenzothiazole/2-amino-4-chlorobenzothiazole-AgNPs, (j) 2-butyne-1,4-diol/2-butyne-1,4-diol-AgNPs, (k) 3-butyn-1-ol/3-butyn-1-ol-AgNPs, (l) heptane-1,7-dioic/ heptane-1,7-dioic-AgNPs.

FTIR Analysis of AgNPs with Organic Ligands

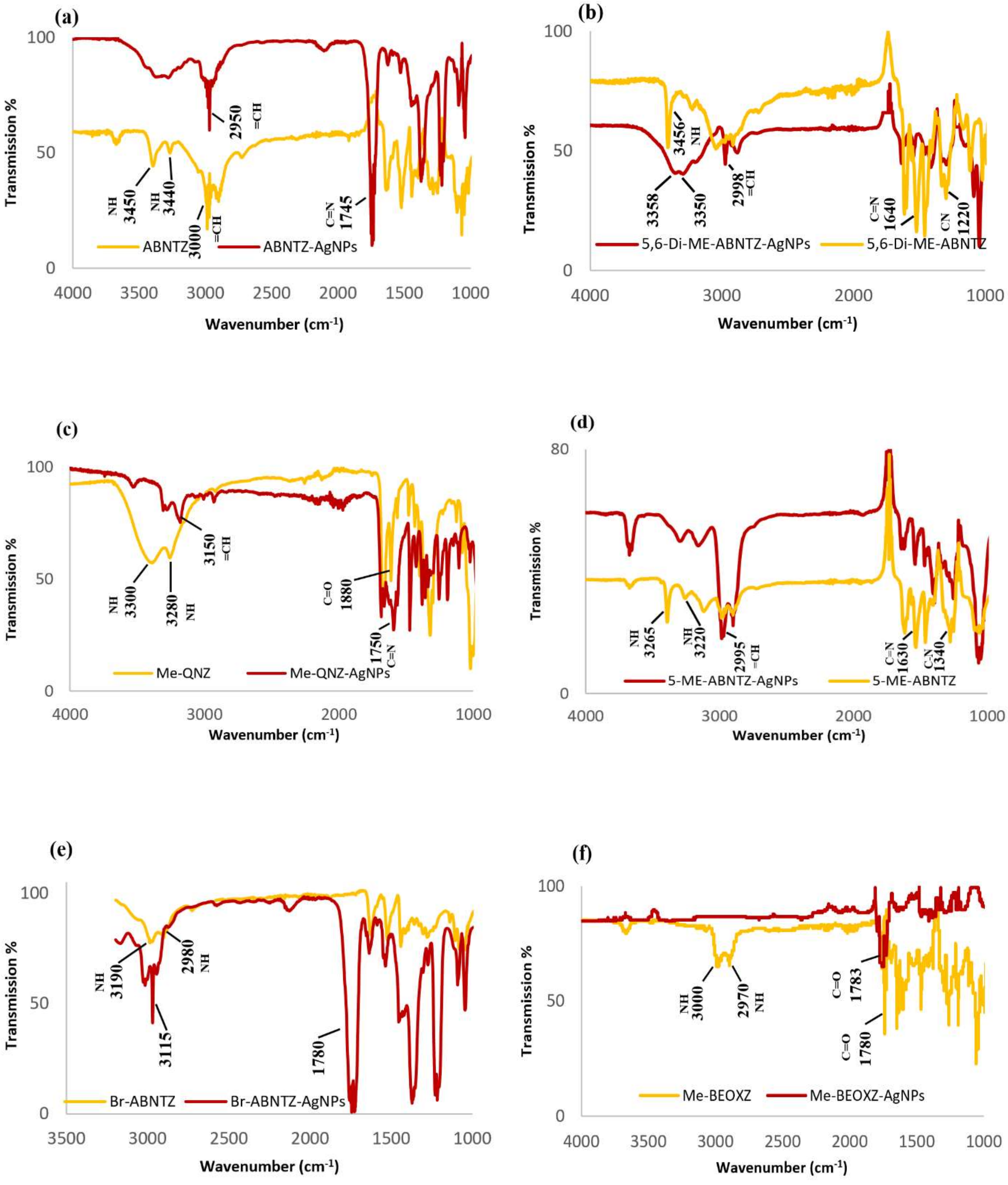

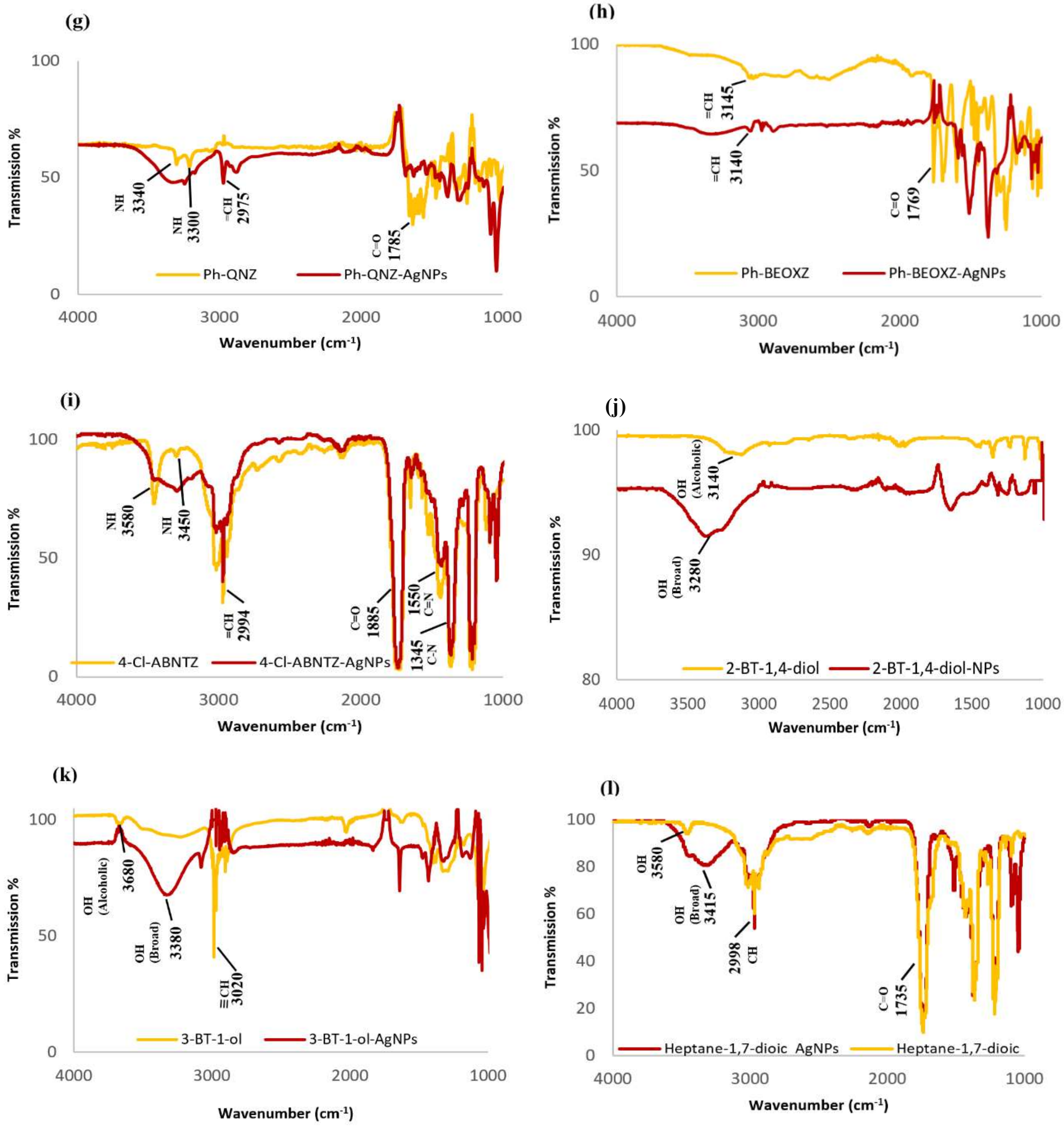

Figure S2. FT-IR analysis of conjugated AgNPs (Red line) with its organic ligand (Yellow line) (a) 2-aminobenzothiazole/2-aminobenzothiazole-AgNPs, (b) 5,6,-dimethyl-2-aminobenzothiazole/5,6,-dimethyl-2-aminobenzothiazole-AgNPs, (c) 3-amino-2-methyl-4(3*H*)-quinazolinone/3-amino-2-methyl-4(3*H*)-quinazolinone-AgNPs, (d) 5-methyl-2-aminobenzothiazole/5-methyl-2-aminobenzothiazole-AgNPs, (e) 2-amino-6-bromobenzothiazole/2-amino-6-bromobenzothiazole-AgNPs, (f) 2-methyl-4*H*-3,1-benzoxazin-4-one/2-methyl-4*H*-3,1-benzoxazin-4-one-AgNPs, (g) 3-amino-2-phenyl-4(3*H*)-quinazolinone/3-amino-2-phenyl-4(3*H*)-quinazolinone-AgNPs, (h) 2-phenyl-4*H*-3,1-benzoxazin-4-one/2-phenyl-4*H*-3,1-benzoxazin-4-one-AgNPs, (i) 2-amino-4-chlorobenzothiazole/2-amino-4-chlorobenzothiazole-AgNPs, (j) 2-butyne-1,4-diol/2-butyne-1,4-diol-AgNPs, (k) 3-butyn-1-ol/3-butyn-1-ol-AgNPs, (l) heptane-1,7-dioic/ heptane-1,7-dioic-AgNPs.

EDAX Analysis of AgNPs

(a)

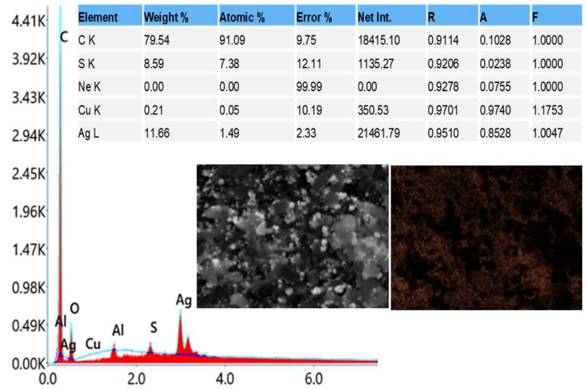

Element	Weight %	Atomic %	Error %	Net Int.	R	A	F
C K	79.54	91.09	9.75	18415.10	0.9114	0.1028	1.0000
S K	8.59	7.38	12.11	1135.27	0.9206	0.0238	1.0000
Ne K	0.00	0.00	99.99	0.00	0.9278	0.0755	1.0000
Cu K	0.21	0.05	10.19	350.53	0.9701	0.9740	1.1753
Ag L	11.66	1.49	2.33	21461.79	0.9510	0.8528	1.0047

(b)

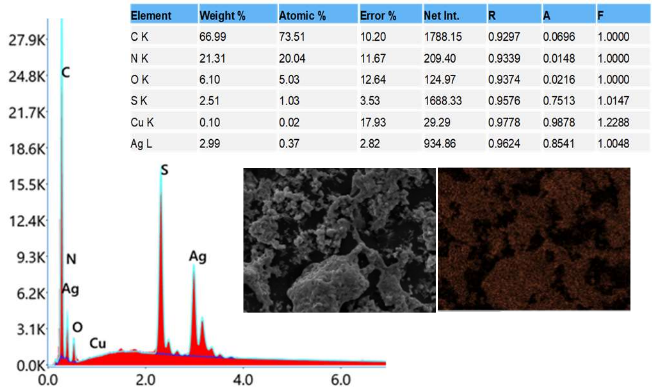

Element	Weight %	Atomic %	Error %	Net Int.	R	A	F
C K	66.99	73.51	10.20	1788.15	0.9297	0.0696	1.0000
N K	21.31	20.04	11.67	209.40	0.9339	0.0148	1.0000
O K	6.10	5.03	12.64	124.97	0.9374	0.0216	1.0000
S K	2.51	1.03	3.53	1688.33	0.9576	0.7513	1.0147
Cu K	0.10	0.02	17.93	29.29	0.9778	0.9878	1.2288
Ag L	2.99	0.37	2.82	934.86	0.9624	0.8541	1.0048

(c)

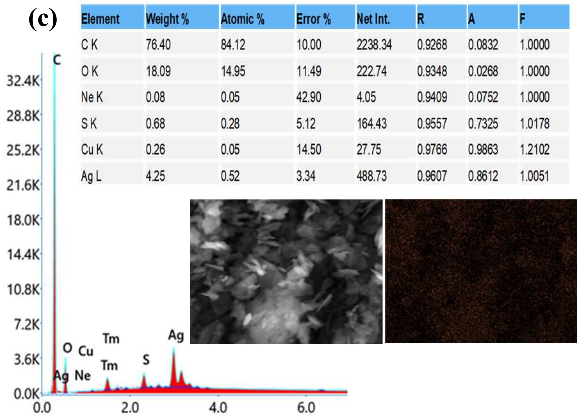

Element	Weight %	Atomic %	Error %	Net Int.	R	A	F
C K	76.40	84.12	10.00	2238.34	0.9268	0.0832	1.0000
O K	18.09	14.95	11.49	222.74	0.9348	0.0268	1.0000
Ne K	0.08	0.05	42.90	4.05	0.9409	0.0752	1.0000
S K	0.68	0.28	5.12	164.43	0.9557	0.7325	1.0178
Cu K	0.26	0.05	14.50	27.75	0.9766	0.9863	1.2102
Ag L	4.25	0.52	3.34	488.73	0.9607	0.8612	1.0051

(d)

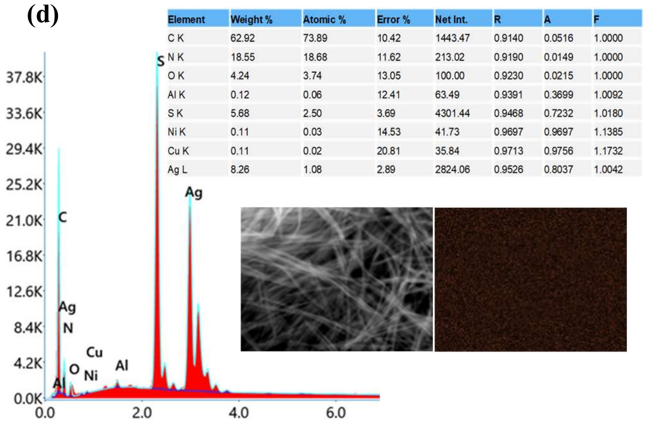

Element	Weight %	Atomic %	Error %	Net Int.	R	A	F
C K	62.92	73.89	10.42	1443.47	0.9140	0.0516	1.0000
N K	18.55	18.68	11.62	213.02	0.9190	0.0149	1.0000
O K	4.24	3.74	13.05	100.00	0.9230	0.0215	1.0000
Al K	0.12	0.06	12.41	63.49	0.9391	0.3699	1.0092
S K	5.68	2.50	3.69	4301.44	0.9468	0.7232	1.0180
Ni K	0.11	0.03	14.53	41.73	0.9697	0.9697	1.1385
Cu K	0.11	0.02	20.81	35.84	0.9713	0.9756	1.1732
Ag L	8.26	1.08	2.89	2824.06	0.9526	0.8037	1.0042

(e)

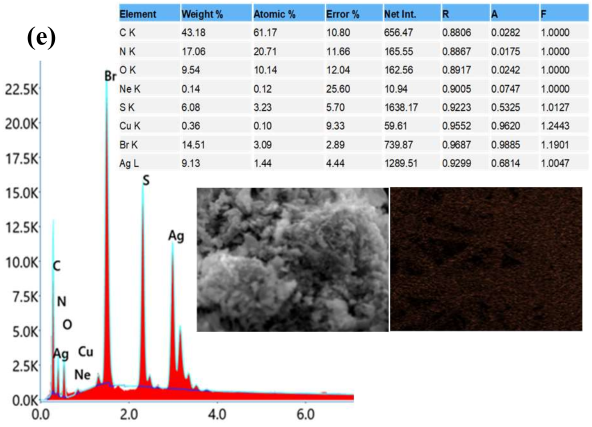

Element	Weight %	Atomic %	Error %	Net Int.	R	A	F
C K	43.18	61.17	10.80	656.47	0.8806	0.0282	1.0000
N K	17.06	20.71	11.66	165.55	0.8867	0.0175	1.0000
O K	9.54	10.14	12.04	162.56	0.8917	0.0242	1.0000
Ne K	0.14	0.12	25.60	10.94	0.9005	0.0747	1.0000
S K	6.08	3.23	5.70	1638.17	0.9223	0.5325	1.0127
Cu K	0.36	0.10	9.33	59.61	0.9552	0.9620	1.2443
Br K	14.51	3.09	2.89	739.87	0.9687	0.9885	1.1901
Ag L	9.13	1.44	4.44	1289.51	0.9299	0.6814	1.0047

(f)

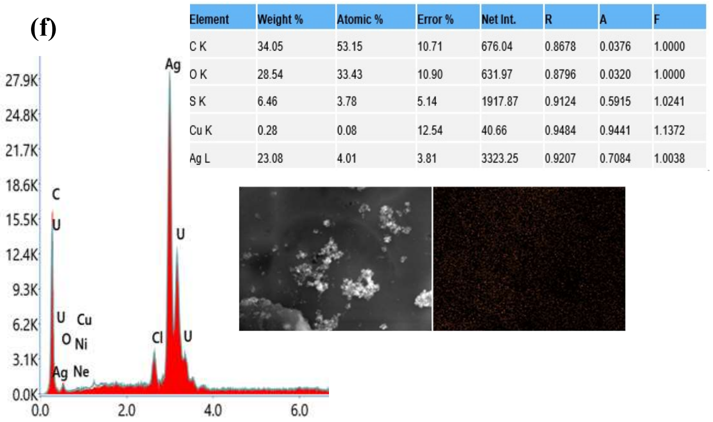

Element	Weight %	Atomic %	Error %	Net Int.	R	A	F
C K	34.05	53.15	10.71	676.04	0.8678	0.0376	1.0000
O K	28.54	33.43	10.90	631.97	0.8796	0.0320	1.0000
S K	6.46	3.78	5.14	1917.87	0.9124	0.5915	1.0241
Cu K	0.28	0.08	12.54	40.66	0.9484	0.9441	1.1372
Ag L	23.08	4.01	3.81	3323.25	0.9207	0.7084	1.0038

(g)

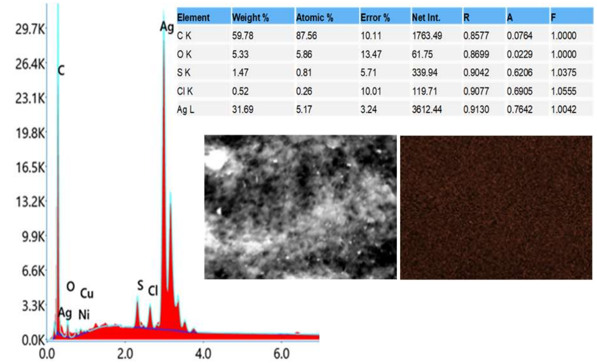

Element	Weight %	Atomic %	Error %	Net Int.	R	A	F
C K	59.78	87.56	10.11	1763.49	0.8577	0.0764	1.0000
O K	5.33	5.86	13.47	61.75	0.8699	0.0229	1.0000
S K	1.47	0.81	5.71	339.94	0.9042	0.6206	1.0375
Cl K	0.52	0.26	10.01	119.71	0.9077	0.6905	1.0555
Ag L	31.69	5.17	3.24	3612.44	0.9130	0.7642	1.0042

(h)

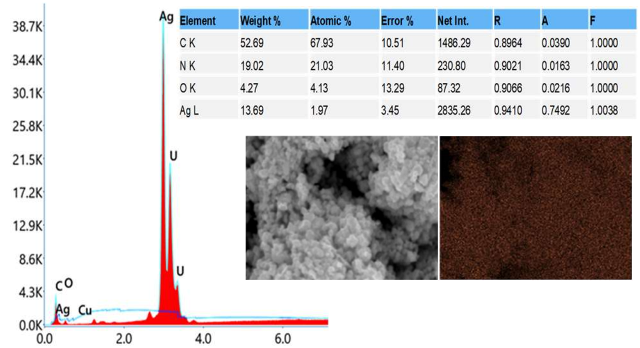

Element	Weight %	Atomic %	Error %	Net Int.	R	A	F
C K	52.69	67.93	10.51	1486.29	0.8964	0.0390	1.0000
N K	19.02	21.03	11.40	230.80	0.9021	0.0163	1.0000
O K	4.27	4.13	13.29	87.32	0.9066	0.0216	1.0000
Ag L	13.69	1.97	3.45	2835.26	0.9410	0.7492	1.0038

(i)

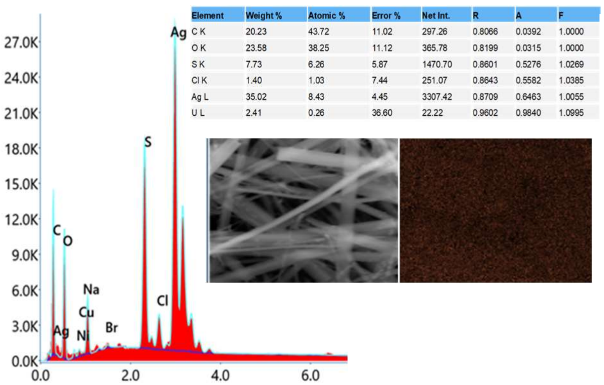

Element	Weight %	Atomic %	Error %	Net Int.	R	A	F
C K	20.23	43.72	11.02	297.26	0.8066	0.0392	1.0000
O K	23.58	38.25	11.12	365.78	0.8199	0.0315	1.0000
S K	7.73	6.26	5.87	1470.70	0.8601	0.5276	1.0269
Cl K	1.40	1.03	7.44	251.07	0.8643	0.5582	1.0385
Ag L	35.02	8.43	4.45	3307.42	0.8709	0.6463	1.0055
U L	2.41	0.26	36.60	22.22	0.9602	0.9840	1.0995

(j)

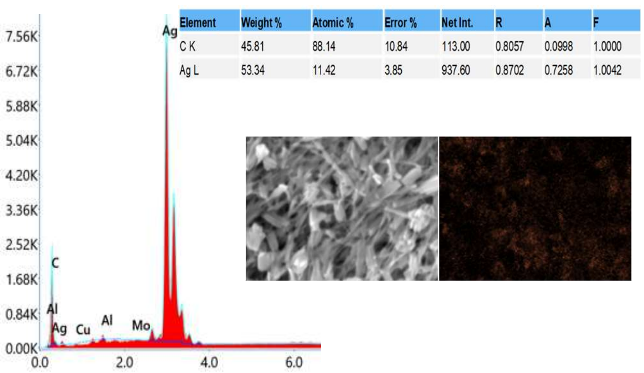

Element	Weight %	Atomic %	Error %	Net Int.	R	A	F
C K	45.81	88.14	10.84	113.00	0.8057	0.0998	1.0000
Ag L	53.34	11.42	3.85	937.60	0.8702	0.7258	1.0042

(k)

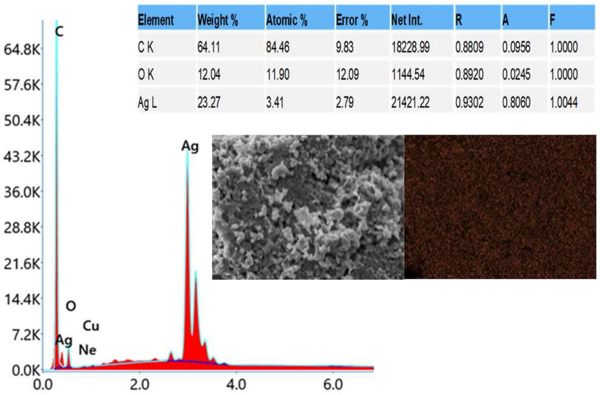

Element	Weight %	Atomic %	Error %	Net Int.	R	A	F
C K	64.11	84.46	9.83	18228.99	0.8809	0.0956	1.0000
O K	12.04	11.90	12.09	1144.54	0.8920	0.0245	1.0000
Ag L	23.27	3.41	2.79	21421.22	0.9302	0.8060	1.0044

(l)

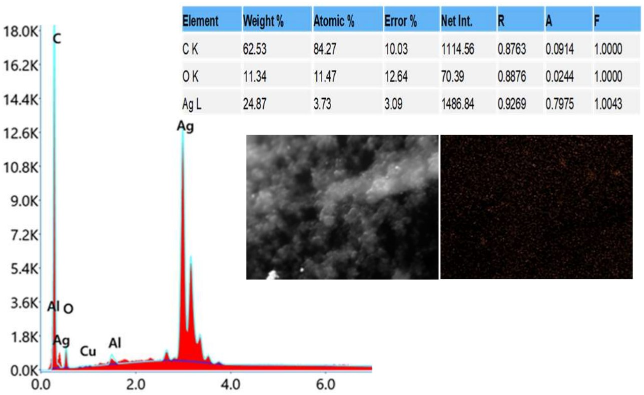

Element	Weight %	Atomic %	Error %	Net Int.	R	A	F
C K	62.53	84.27	10.03	1114.56	0.8763	0.0914	1.0000
O K	11.34	11.47	12.64	70.39	0.8876	0.0244	1.0000
Ag L	24.87	3.73	3.09	1486.84	0.9269	0.7975	1.0043

Figure S3. EDX analysis of each conjugated silver nanoparticles (a) 2-aminobenzothiazole-AgNPs, (b) 5,6,-dimethyl-2-aminobenzothiazole-AgNPs, (c) 3-Amino-2-methyl-4(3*H*)-quinazolinone-AgNPs, (d) 5-Methyl-2-aminobenzothiazole-AgNPs, (e) 2-Amino-6-bromobenzothiazole-AgNPs, (f) 2-methyl-4*H*-3,1-benzoxazin-4-one-AgNPs, (g) 3-Amino-2-phenyl-4(3*H*)-quinazolinone-AgNPs, (h) 2-Phenyl-4*H*-3,1-benzoxazin-4-one-AgNPs, (i) 2-Amino-4-chlorobenzothiazole-AgNPs, (j) 2-Butyne-1,4-diol-AgNPs, (k) 3-Butyn-1-ol-AgNPs, (l) Heptane-1,7-dioic-AgNPs.

Size Distribution Histogram of AgNPs

(a)

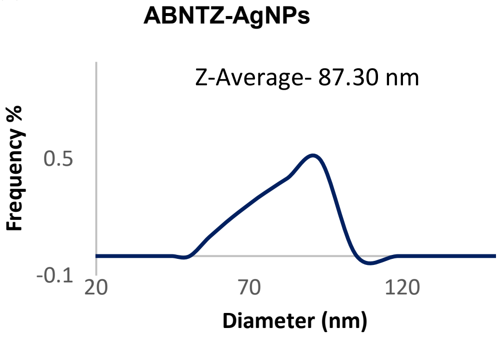

(b)

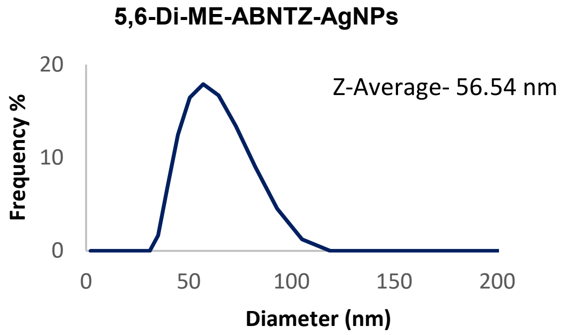

(c)

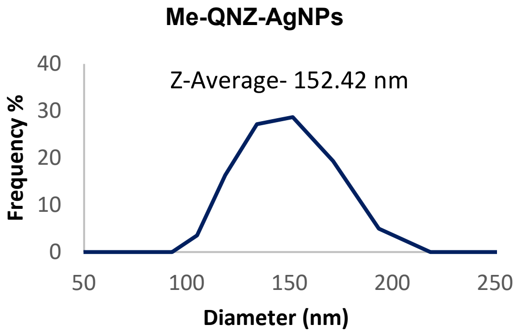

(d)

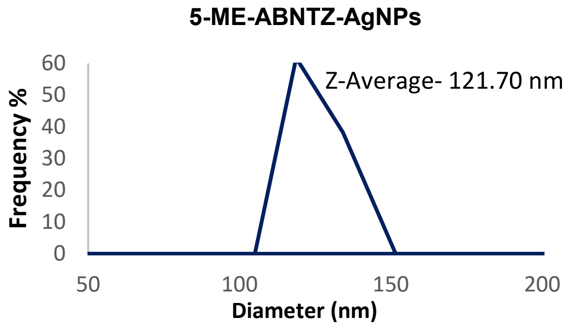

(e)

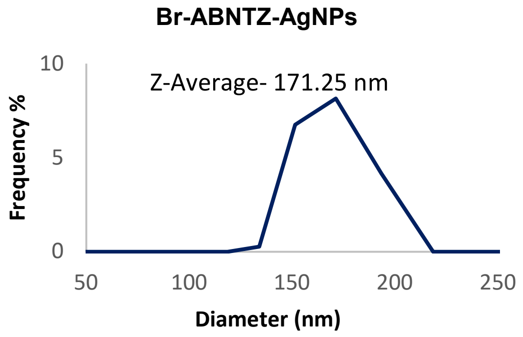

(f)

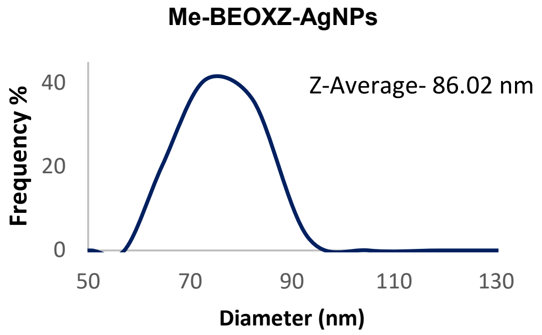

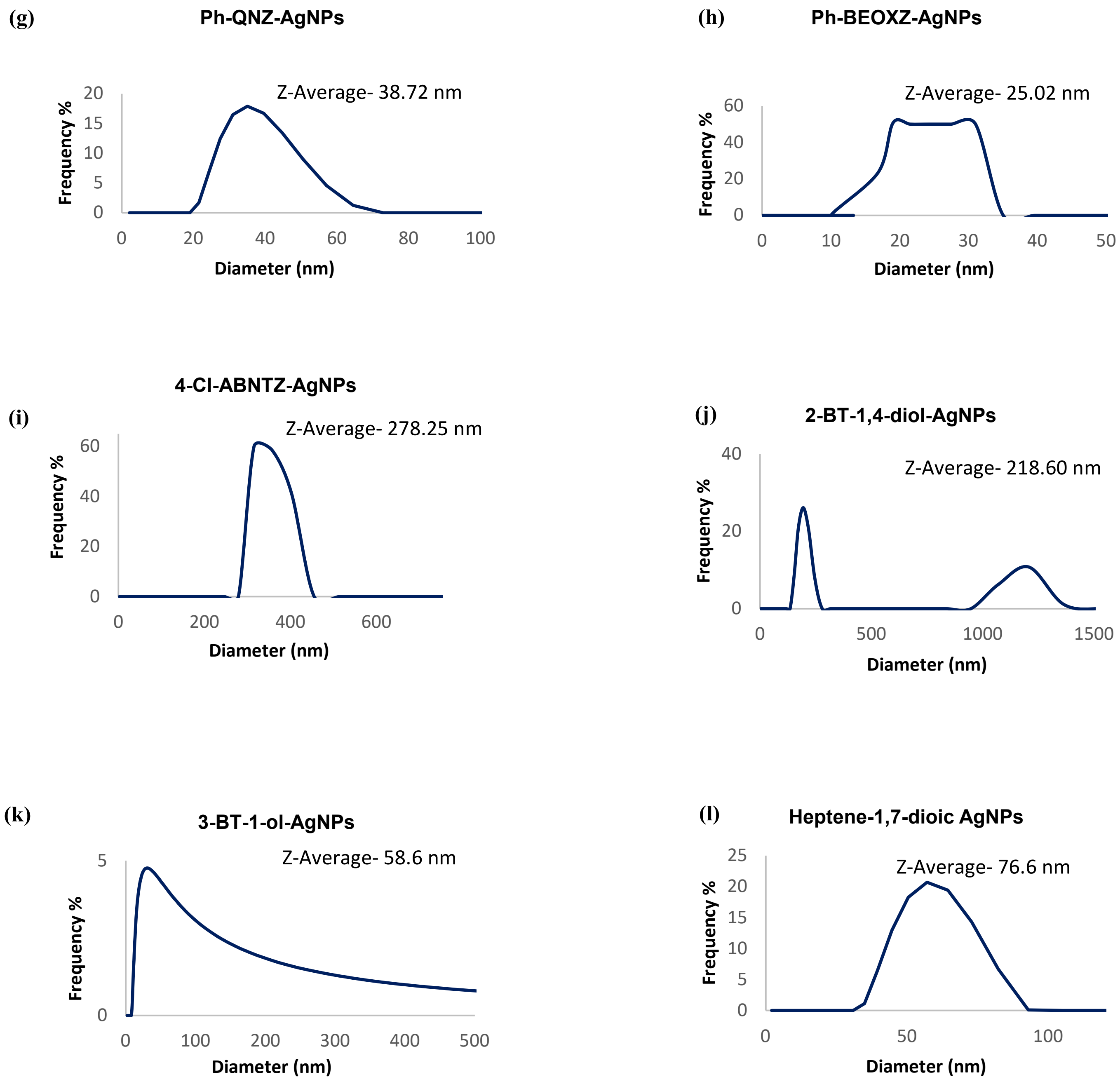

Figure S4. Size distribution histogram (a) 2-aminobenzothiazole-AgNPs, (b) 5,6,-dimethyl-2-aminobenzothiazole-AgNPs, (c) 3-Amino-2-methyl-4(3*H*)-quinazolinone-AgNPs, (d) 5-Methyl-2-aminobenzothiazole-AgNPs, (e) 2-Amino-6-bromobenzothiazole-AgNPs, (f) 2-methyl-4*H*-3,1-benzoxazin-4-one-AgNPs, (g) 3-Amino-2-phenyl-4(3*H*)-quinazolinone-AgNPs, (h) 2-Phenyl-4*H*-3,1-benzoxazin-4-one-AgNPs, (i) 2-Amino-4-chlorobenzothiazole-AgNPs, (j) 2-Butyne-1,4-diol-AgNPs, (k) 3-Butyn-1-ol-AgNPs, (l) Heptane-1,7-dioic-AgNPs. The nanoparticles showed diameter in the range of 25–278 nm.

Zeta Potential Analysis of AgNPs

(a)

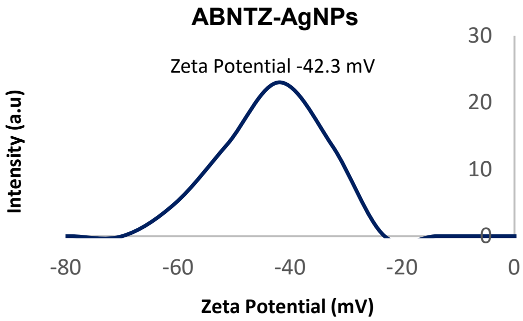

(b)

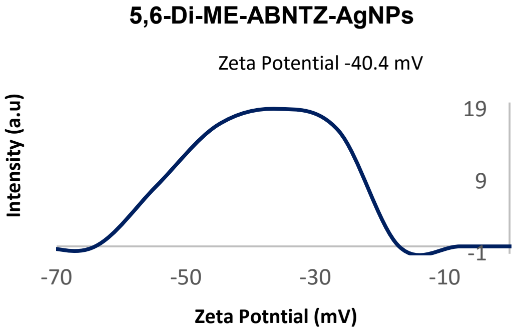

(c)

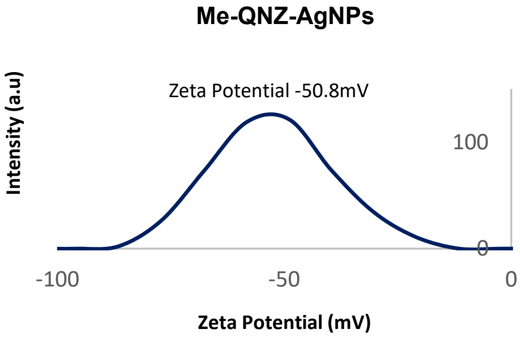

(d)

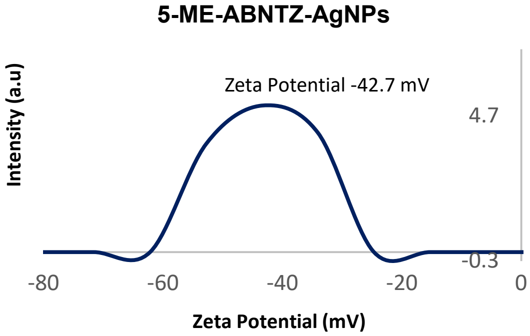

(e)

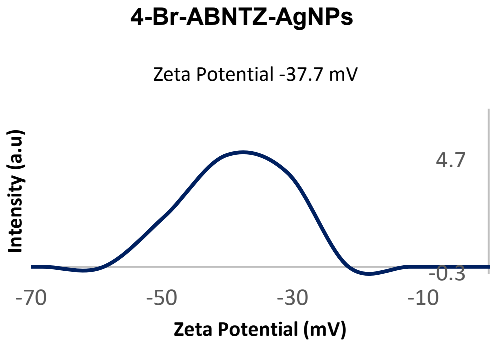

(f)

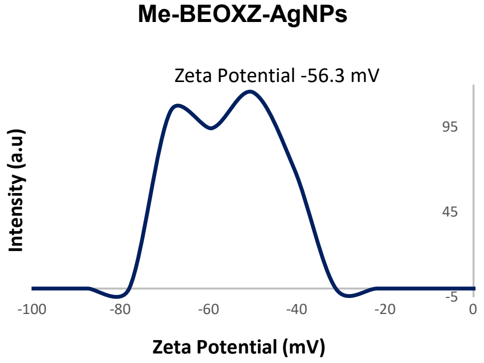

(g)

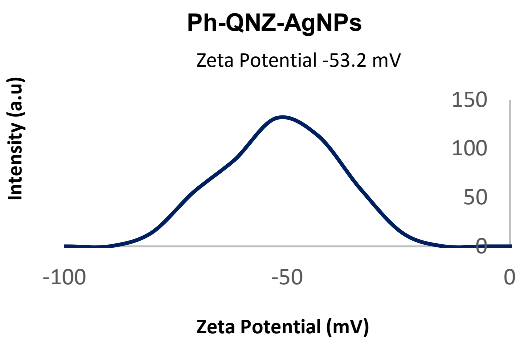

(h)

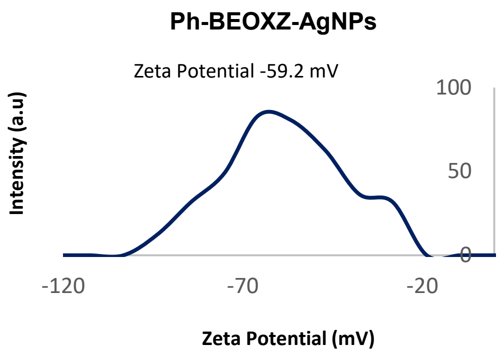

(i)

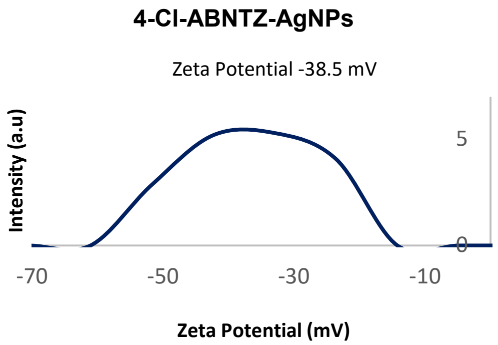

(j)

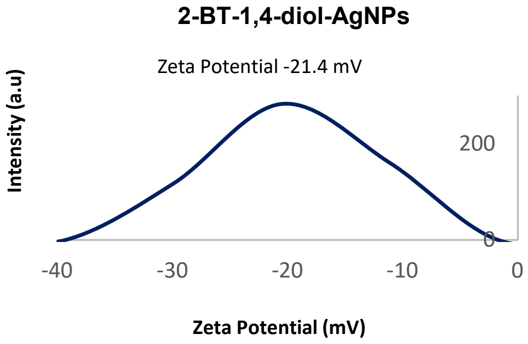

(k)

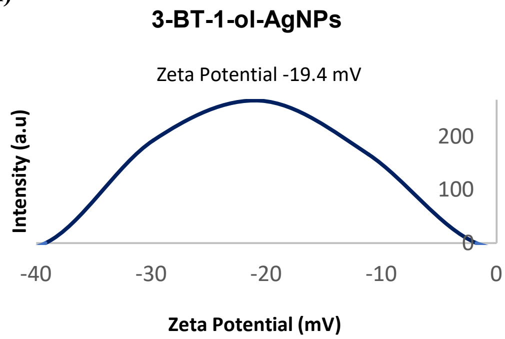

(l)

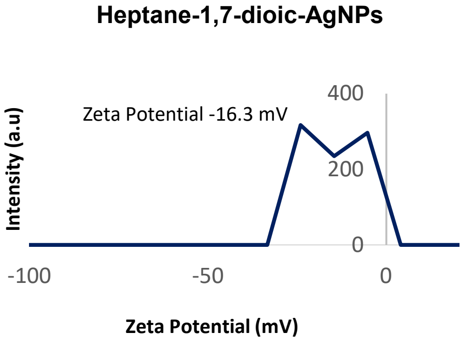

Figure S5. zeta potential plots of (a) 2-Aminobenzothiazole-AgNPs, (b) 5,6-Dimethyl-2-aminobenzothiazole-AgNPs, (c) 3-Amino-2-methyl-4(3*H*) quinazolinone-AgNPs, (d) 5-Methyl-2-aminobenzothiazole-AgNPs, (e) 2-Amino-6-bromobenzothiazole-AgNPs, (f) 2-methyl-4*H*-3,1-benzoxazin-4-one-AgNPs, (g) 3-Amino-2-phenyl-4(3*H*)-quinazolinone-AgNPs, (h) 2-Phenyl-4*H*-3,1-benzoxazin-4-one-AgNPs, (i) 2-Amino-4-chlorobenzothiazole-AgNPs, (j) 2-Butyne-1,4-diol-AgNPs, (k) 3-Butyn-1-ol-AgNPs, (l) Heptane-1,7-dioic-AgNPs.

Antibacterial Assay

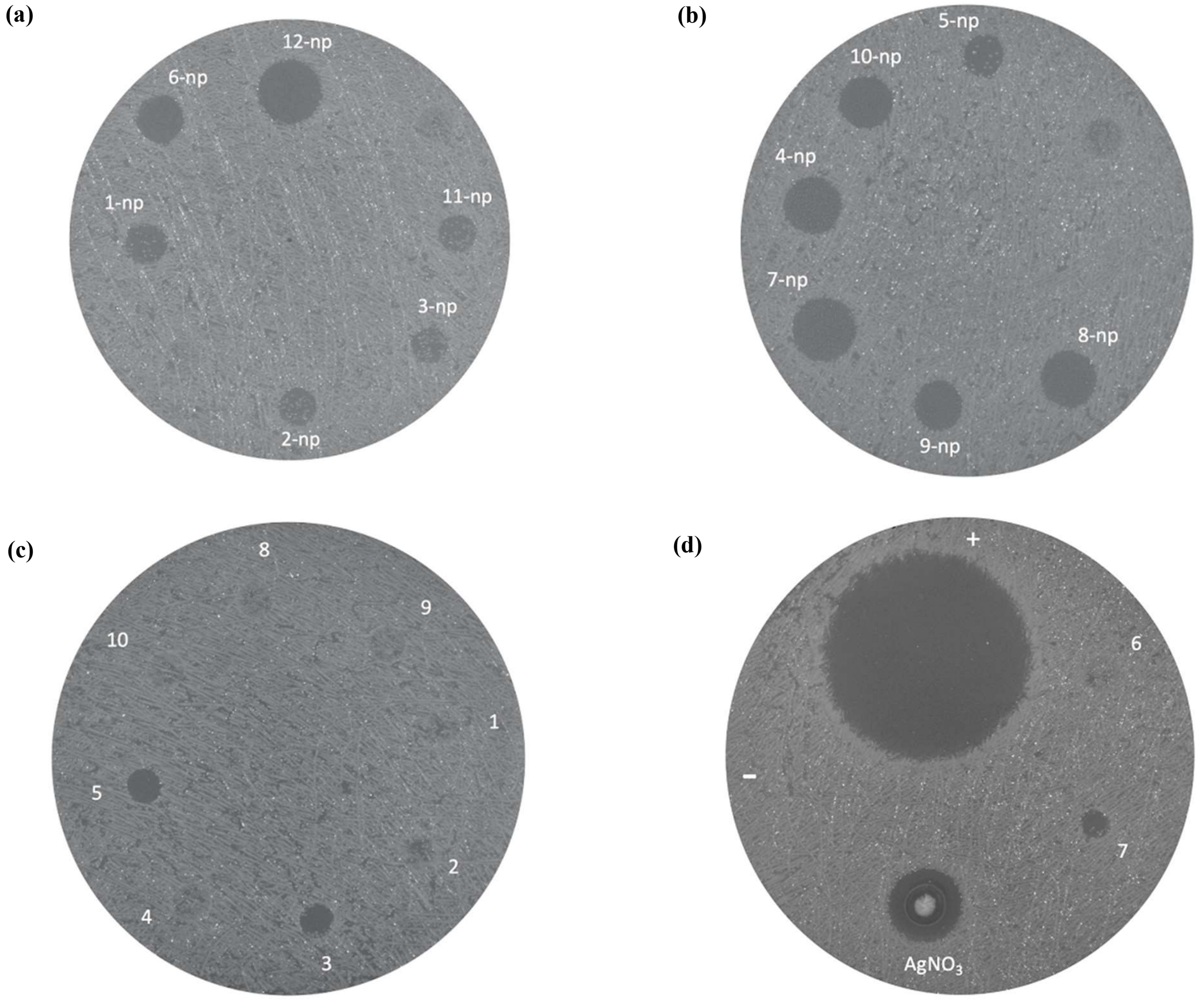

Figure S6. Antibacterial activity against E. coli was measured using a zone of inhibition assay for AgNPs and their corresponding organic ligands (images were taken with a BioRad imager on the black and white setting). Dark circles indicate an absence of bacterial growth on the agar plate. Particles 1-np, 2-np, 3-np, 4-np, 5-np, 6-np, 7-np, 8-np, 9-np, 10-np, 11-np, and 12-np are shown on plates a and b. The zones of inhibition of organic ligands 1, 2, 3, 4, 5, 6, 7, 8, 9, and 10 are shown on plates c and d. Additional controls are (+) ampicillin (100 mg/mL), (-) ultra-pure H2O, and AgNO3 dissolved in ethanol (1 mg/mL) on plate d.
